# Coupling the folding of a β-hairpin with chelation-enhanced luminescence of Tb(iii) and Eu(iii) ions for specific sensing of a viral RNA†
†Electronic supplementary information (ESI) available: Peptide and lanthanide probe synthesis, luminescence experiments, experimental description of the transfection and multiplex experiments. See DOI: 10.1039/c5sc04501k


**DOI:** 10.1039/c5sc04501k

**Published:** 2016-01-11

**Authors:** Cristina Penas, José L. Mascareñas, M. Eugenio Vázquez

**Affiliations:** a Centro Singular de Investigación en Química Biolóxica e Materiais Moleculares (CIQUS) , Departamento de Química Orgánica , Universidade de Santiago de Compostela , 15782 Santiago de Compostela , Spain . Email: joseluis.mascarenas@usc.es ; Email: eugenio.vazquez@usc.es

## Abstract

Rational modification of a natural RNA-binding peptide with a lanthanide EDTA chelator, and a phenanthroline ligand yields a highly selective luminescent sensor. The sensing mechanism relies on the RNA-triggered folding of the peptide into a β-hairpin, which promotes the coordination of the phenanthroline sensitizer, and the efficient sensitization of complexed lanthanide ions.

## Abstract 2

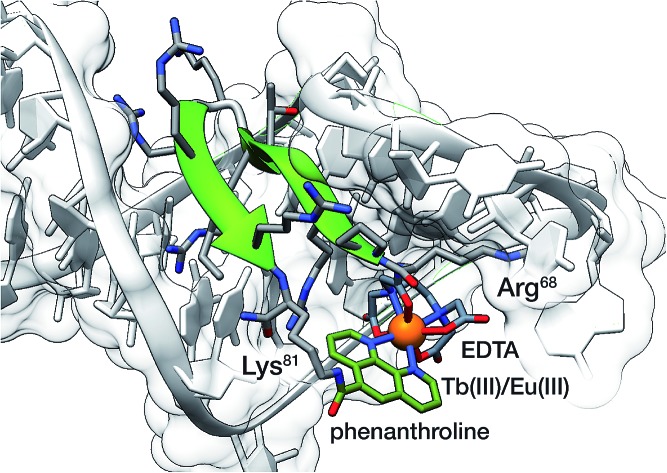
Rational modification of a natural RNA-binding peptide with a lanthanide EDTA chelator, and a phenanthroline ligand yields a highly selective luminescent sensor.

## Introduction

Fluorescence spectroscopy has transformed the study of biological systems by allowing the straightforward visualization of molecular events with extraordinary selectivity and sensitivity.^1^ The success of luminescent techniques relies on the availability of probes able to respond to specific biochemical inputs.^2^ In addition to organic fluorophores, lanthanide ions are privileged emissive species that display unique properties (*e.g.*, narrow emission bands, and extremely long excited state lifetimes) that make them particularly attractive for luminescent sensing.^3^ In combination with peptides, lanthanides are powerful reporters that have been used to detect specific biomolecules,^4^,^5^ and monitor diverse biological processes.^6^,^7^ These luminescent sensors are typically based on the modulation of the energy transfer process from a nearby chromophore—the sensitization process—which mediates the efficient population of the excited states of the lanthanide ions.^8^ We have ourselves used this strategy to develop various lanthanide sensors that rely on the coupling of specific recognition events with the folding of short α-helices.^4^,^9^ Unfortunately, these sensors suffer from considerable background emission resulting from the restrictions imposed by the α-helical platform, that forces the antenna (tryptophan) and the lanthanide (terbium) ions to be placed in close proximity (relative positions *i*, *i* + 4), even in the unbound form. Besides, the folding of α-helices represents only one particular case of structural rearrangements coupled to biomolecular recognition processes, and a more general strategy applicable to the study of other conformational changes would be highly desirable.

**Fig. 1:**
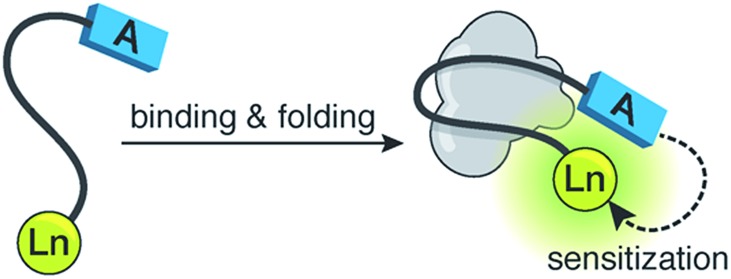
Proposed conformational sensing mechanism.

Herein, we report a new sensing strategy based on the folding of a random coil peptide into a β-hairpin, which allows the introduction of the sensitizing antenna and the lanthanide ion at distant positions in the sequence, thus ensuring low intrinsic emission in absence of the target;^10^ the large conformational changes experienced by the peptide upon folding give rise to a large increase in the lanthanide emission, thus ensuring high sensitivity (Fig. 1). Given that many biomolecular recognition processes involve related folding events,^11^ this strategy should be easily adapted to monitor different biological processes.

The viability of this strategy is demonstrated with the detection of the bovine immunodeficiency virus (BIV) RNA transactivation response element (*TAR*), a secondary RNA structure that is recognized with high affinity and high specificity by a 14-amino acid arginine-rich fragment from the **Tat** transcriptional activator.^12^**Tat** is a sequence-specific adaptor protein that directs the cellular transcription machinery to the viral RNA, thus promoting transcription elongation.^13^ Furthermore, the BIV **Tat** is closely related to its human immunodeficiency virus (HIV) counterpart,^14^ and therefore represents an attractive model to study HIV transactivation.

## Results and discussion

**Fig. 2:**
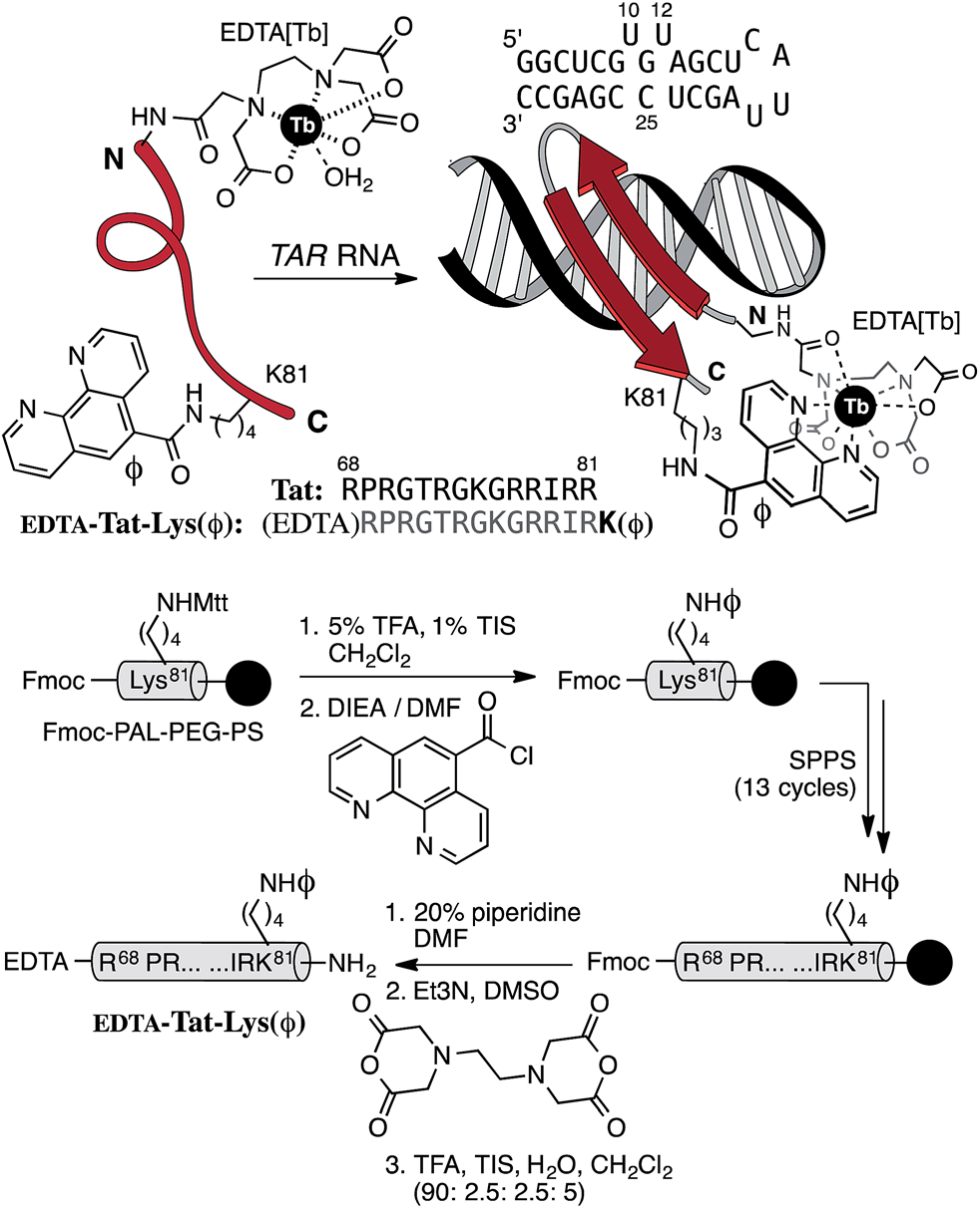
Top. Outline of the sensing mechanism: the folding of the peptide upon binding to the target *TAR* RNA induces the coordination of the phenanthroline antenna to the EDTA[Tb] complex. Sequences of **EDTA-Tat-Lys(φ)**, and the natural **Tat** peptide, as well as the structure of the *TAR* RNA hairpin are shown. Bottom: Key steps in the synthesis of the probe: orthogonal deprotection and derivatization of the C-terminal Lys(Mtt), and N-terminal derivatization with EDTA. φ = 1,10-Phenanthroline-5-carbonyl group.

Based on the solution structure of the peptide **Tat** bound to the BIV *TAR* hairpin RNA,^15^ as well as on mutational studies that identified the essential residues for high-affinity binding,^16^ we envisioned that extending the N-terminus of the **Tat** core sequence (Arg^68^ to Arg^81^) with a Trp residue, and introducing a DOTA[Tb] complex at the C-terminus should result in a metallopeptide probe that, upon binding to its target *TAR* RNA, would fold into a β-hairpin, placing both the DOTA[Tb] complex and the sensitizing indole ring of the Trp close to each other.^17^ Unfortunately, although the designed peptide was weakly emissive by itself, and displayed efficient RNA binding, addition of the target BIV *TAR* RNA did not induce any increase in its luminescence, likely because the excited state of the Trp is rapidly quenched by the RNA bases (most likely G11 and G19 close to the Trp in the bound state) before the sensitization takes place (see details in the ESI†).^18^ Straightforward modifications of that design, such as the addition of spacer residues between the Trp and the DOTA[Tb], switching their positions in the peptide chain, or using other chromophores, led in all cases to derivatives that were also spectroscopically unresponsive (see details in the ESI†). In light of these results, we reconsidered our initial design, and envisioned that forcing the direct complexation of the sensitizing chromophore to the lanthanide might favor the sensitization over the competitive quenching. Thus, we designed the peptide probe **EDTA[Tb]-Tat-Lys(φ)**, featuring an EDTA chelator at its N-terminus (in place of the original DOTA),^19^ and a phenanthroline coordinating antenna (φ) attached to the side chain of a Lys residue in place of the C-terminal Arg^81^ (Fig. 2, top).^20^

The target peptide **EDTA-Tat-Lys(φ)** was assembled following standard Fmoc/*t*Bu solid-phase peptide synthesis procedures.^21^ The phenanthroline unit was connected to the side chain of an orthogonally-deprotected Lys(Mtt) residue while still attached to the solid support.^22^ After synthesizing the rest of the peptide sequence, the N-terminal amine (Arg^68^) was acylated with EDTA dianhydride (Fig. 2, bottom). The final peptide was purified by reverse-phase HPLC and identified by MS (see details in the ESI†).

**Fig. 3:**
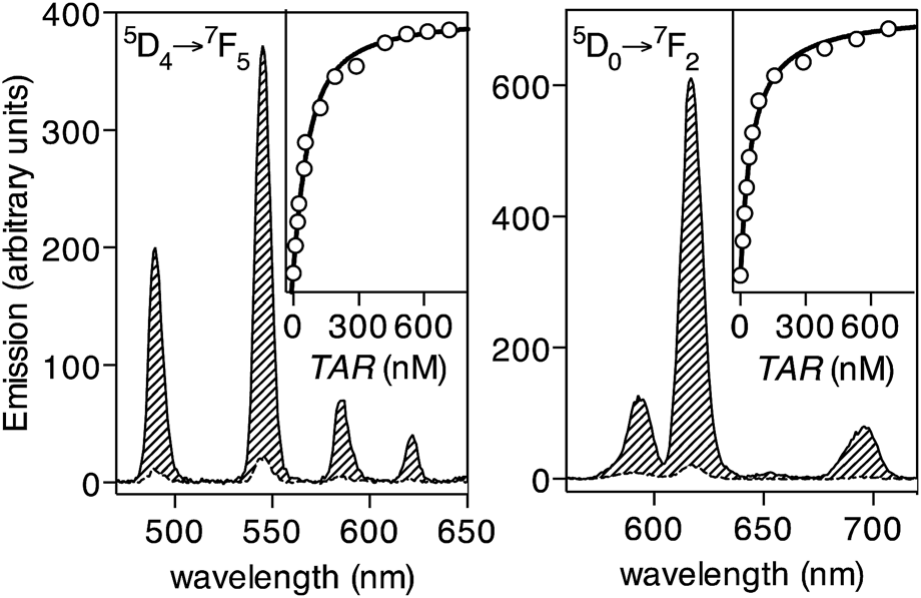
Time-gated emission spectra of **EDTA[Tb]-Tat-Lys(φ)** (left) and **EDTA[Eu]-Tat-Lys(φ)** (right) upon excitation of the phenanthroline antenna at 300 nm. The spectra of the free probes are shown as dashed lines, and the those at saturating concentration of the *TAR* RNA as solid lines. Insets show representative titrations of the Tb(iii) and Eu(iii) probes (emission at 545 nm and 615, respectively) with increasing concentrations of the *TAR* RNA. The lines represent the best fits according to a 1 : 1 binding model. Spectra were measured at a 50 nM concentration of the peptide probes in 10 mM HEPES buffer, 100 mM NaCl, pH 7.6 (0.2 ms delay).

Having at hand the desired peptide probe, we studied the effect of the *TAR* RNA in the emission of its Tb(iii) complex. As expected, the **EDTA[Tb]-Tat-Lys(φ)** metallopeptide, formed *in situ* by incubating the peptide **EDTA[Tb]-Tat-Lys(φ)** with 1 eq. of TbCl_3_, does not show significant emission from the Tb(iii) ion upon irradiation of the phenanthroline antenna at 300 nm (Fig. 3, left, dashed trace, *Φ*[Tb] ≈ 0.010).^23^ Gratifyingly, incubation of this probe with increasing concentrations of the target BIV *TAR* RNA hairpin gives rise to a large increase in the luminescent emission bands of the Tb(iii) ion (about 12-fold increase, *Φ*[Tb]/*TAR* ≈ 0.136).^24^ This is consistent with the folding of the **Tat** peptide core into a hairpin structure, and chelation of the terbium ion by the phenanthroline antenna upon binding to the RNA (Fig. 3, left, solid line). A titration experiment using the emission intensity of the hypersensitive band at 545 nm, corresponding to the ^5^D_4_ → ^7^F_5_ transition of the Tb(iii) ion,^25^ allowed to calculate the dissociation constant of the probe *K*_D_ ≈ 57 nM (Fig. 3, inset in the left graph), which is in agreement with the reported affinity of the natural **Tat** peptide for this hairpin RNA (*K*_D_[**Tat**] in the range of 50–100 nM).^16^ The calculated detection limit is ≈5 nM, which is well below the typical viral RNA concentration in infected cells.^26^ As expected, the probe is highly selective for the *TAR* RNA hairpin, and does not increase its emission in the presence of other non-target RNAs or DNAs; specific *TAR* RNA recognition by the **EDTA[Tb]-Tat-Lys(φ)** peptide is also demonstrated by EMSA and CD experiments (see details in the ESI†). Additionally, the phenanthroline antenna can also efficiently sensitize Eu(iii) ions, and thus the analogous **EDTA[Eu]-Tat-Lys(φ)** probe also responds to the addition of the target *TAR* RNA with a large (30-fold) increase in the ^5^D_0_ → ^7^F_2_ emission band of Eu(iii) at *c.a.* 615 nm (Fig. 3, right). The chelated **EDTA[Eu]-Tat-Lys(φ)** displays higher emission intensity than the bound Tb(iii) probe (*Φ*[Eu] ≈ 0.0046; *Φ*[Eu]/*TAR* ≈ 0.165, over 30-fold increase).^20^,^28^ The lower sensitivity of the probe with Tb(iii) might arise from the small energy gap between the triplet state of the phenanthroline antenna and the emitting ^5^D_4_ level in the metal ion, which favors efficient sensitization, but also allows significant energy back-transfer to the chromophore,^27^ thus reducing the overall sensitization efficiency.

Luminescence lifetime experiments with the **EDTA[Tb]-Tat-Lys(φ)** probe in D_2_O and H_2_O allowed us to calculate the number of inner-sphere water molecules bound to the metal center (*q*),^29^ which turned out to be *q* ≈ 1 for the probe in absence of its target RNA, and *q* ≈ 0 at saturating concentrations of *TAR* RNA. These values are in agreement with the reported number of water molecules coordinating the EDTA[Tb] complex, and support the RNA-promoted chelation of the ion upon folding of the peptide chain.^30^,^31^ Interestingly, it is known that OH oscillators in bound water molecules are efficient quenchers of lanthanide emission, and thus the displacement of the inner-sphere water of the EDTA complex upon RNA binding could also contribute to the large increase in emission observed upon folding and chelation.

**Fig. 4:**
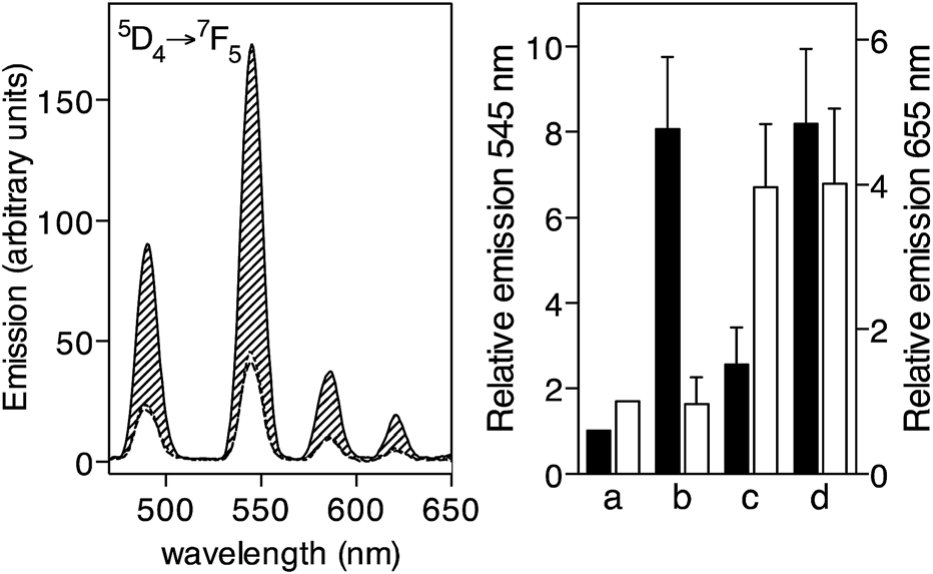
Left: Time-gated emission spectra of 150 nM **EDTA[Tb]-Tat-Lys(φ)** in complete cell lysates. Solid line, cells transfected with a 2 μg mL^–1^ solution of the target *TAR* RNA; dotted line, cells transfected with a 2 μg mL^–1^ solution of the non-target *boxB* RNA (RNA); dashed line, cells without no exogenous RNA transfected. Right: Relative luminescence of a mixture of two RNA sensors: the **P22-N^W^[Tb]***boxB* hairpin RNA probe (Tb emission at 545 nm, black bars) and the orthogonal **EDTA[Eu]-Tat-Lys(φ)***TAR* RNA probe (Eu emission at 615 nm, white bars): (a) normalized emission of a solution containing 50 nM of both probes in HEPES buffer, 100 mM NaCl, pH 7.5 (0.2 ms delay); (b) emission of the solution in (a) in the presence of 50 nM of the *boxB* hairpin RNA; (c) solution in (a) in the presence of 50 nM of *TAR* RNA; (d) same solution in (a) in the presence of a mixture of *boxB* and *TAR* RNAs (both at 50 nM).

Importantly, the **EDTA[Tb]-Tat-Lys(φ)** probe specifically detects intracellular *TAR* RNA, displaying a large increase in its luminescence when added to cell lysates obtained from *TAR* RNA transfected HeLa cells (Fig. 4, left, solid line), while no increase in its emission was detected upon incubation with lysates from cells transfected with a non-target RNA (Fig. 4, left, dotted line), as well as with control lysates from untransfected HeLa cells (Fig. 4, left, dashed line). This experiment also demonstrates the advantages of the long luminescence lifetime of the lanthanide ions that allow time-gated conditions to isolate the emission of the lanthanide ions from the short-lived fluorescence of the biological background.^32^

Finally, we also demonstrated the potential of this newly developed probe, **EDTA[Eu]-Tat-Lys(φ)**, for multiplex RNA sensing in combination with **P22-N^W^[Tb]**, a previously described probe targeting the *boxB* RNA hairpin.9*b* Towards this end, we first demonstrated that the two probes, **P22-N^W^[Tb]**, and **EDTA[Eu]-Tat-Lys(φ)**, were capable of independently signaling the presence of their target hairpin RNAs, *boxB* and *TAR*, respectively. Thus, incubation of a 50 nM mixture of both sensors with *boxB* results in a large increase of the Tb(iii) emission at 545 nm, resulting from the α-helical folding of the **P22-N^W^[Tb]** probe (Fig. 4, right, a and b),^33^ whereas addition of *TAR* RNA produced a large and selective increase of the **EDTA[Eu]-Tat-Lys(φ)** emission at 655 nm (Fig. 4, right, a and c). As expected, the addition of both RNA hairpins gives rise to emission increases in both probes, similar to those previously observed (Fig. 4, right, d).^34^

## Conclusions

In summary, we have developed a new luminescence sensing strategy that takes advantage of the large conformational changes associated to recognition processes involving the folding of a β-hairpin for the effective modulation of lanthanide sensitization processes. Importantly, we found that using a lanthanide-coordinating antenna (phenanthroline) results in sensors that display both excellent dissociation constant and detection limit, with demonstrated bioanalytical applications. Finally, the modularity of this approach should allow its straightforward application for the development of sensors for monitoring other processes involving related folding events.

## Supplementary Material

Supplementary information
