# Supplementary material for: Coupling the folding of a β-hairpin with chelation-enhanced luminescence of Tb(iii) and Eu(iii) ions for specific sensing of a viral RNA
Source: Chem Sci. 2016 Jan 11;7(4):2674–8. doi: 10.1039/c5sc04501k (PMC4898589; doi:10.1039/c5sc04501k)
Supplement: Supplementary file 1 [file SC-007-C5SC04501K-s001.pdf]

Electronic Supplementary Material (ESI) for Chemical Science.
This journal is © The Royal Society of Chemistry 2016

Supporting Information

Coupling the folding of a β-hairpin with chelation-enhanced luminescence of Tb(III) and Eu(III) ions for specific sensing of a viral RNA

Cristina Penas, José L. Mascareñas,* M. Eugenio Vázquez

Centro Singular de Investigación en Química Biolóxica e Materiais Moleculares (CIQUS), Departamento de Química Orgánica. Universidade de Santiago de Compostela, 15782 Santiago de Compostela. Spain.

General 1
General peptide synthesis procedures 2
1. Natural peptide **Tat** 3
1.1. Synthesis *3*
2. Peptide with a tryptophan antenna: **W-Tat-K(DOTA[Tb])** 3
2.1. Design and structure *3*
2.2. Synthesis *4*
2.3. Biophysical assays *6*
Luminescence measurements and EMSA experiments 6
3. Alternative peptides 7
3.1. Design and structures *7*
3.2. Synthesis of small molecular fragments and assembly of peptides *8*
Synthesis of the acridine derivative **1** 8
Synthesis of 1-(carboxymethyl)- 4-methyl-chinolinium bromide (**2**) 10
Synthesis of thiobenzoxazole (**3**) 10
Synthesis of thiazole orange derivative **4** 10
Synthesis of peptides **W-O1Pen-Tat-K(DOTA [Tb])**, **W-G-Tat-K(DOTA [Tb])**, **W-P-Tat-K(DOTA [Tb])** and **W-W-Tat-K(DOTA [Tb])** 11
Synthesis of peptide **(DOTA [Tb])-Tat-W** 11
Synthesis of peptides **Cou-Tat-K(DOTA[Eu])**, **Cou-P_4-Tat-P_4-K(DOTA [Eu])**, **ACR-Tat-K(DOTA [Eu])** and **TO-Tat-K(DOTA [Eu])** 12
3.3. Biophysical assays *15*
Luminescence measurements 15
4. Peptides with phenantroline antennas 18
*4.1. Synthesis of the phenantroline derivative **8*** *18*
4.2. Synthesis of peptide EDTA[Tb]-Tat-Lys(ϕ) *19*
Analysis of EDTA-Tat-Lys(ϕ)/Tb(III) and EDTA-Tat-Lys(ϕ)/Eu(III) mixtures by MS. 20
4.3. Biophysical assays *21*
Luminescence measurements 21
Multicolor detection experiments 24
Determination of luminescence quantum yields 25
Circular Dichroism 26
EMSA experiments 26

General

All reagents were acquired from commercial sources: DMF and TFA were purchased from *Scharlau*, CH_2Cl_2 from *Panreac* and CH_3CN from *Merck*. The rest of the reagents were acquired from *Sigma-Aldrich*. Reactions were monitored by analytical RP-HPLC with an *Agilent 1100* series LC/MS using an *Eclipse* XDB-C_{18} (4.6 × 150 mm, 5 μm) analytical column. Compounds were detected by UV absorption (220, 270, 380 and 495 nm); the standard conditions for analytical RP-HPLC consisted on an isocratic regime during the first 5 min, followed by a linear gradient from 5 to 95% of solvent B for 30 min at a flow rate of 1 mL/min (A: water with 0.1% TFA, B: acetonitrile with 0.1% TFA).

Electrospray Ionization Mass Spectrometry (ESI/MS) of compounds **EDTA-Tat-Lys(ϕ)**/Eu(III) and **EDTA-Tat-Lys(ϕ)**/Tb(III) was performed with a *Bruker Amazon* IT/MS in positive scan mode using direct injection of the peptide-lanthanide solution into the MS.

Purification of compounds was carried out by column chromatography using as stationary phase silica gel 60 SDS type, 230-400 mesh (*Merck*) and using mixtures of MeOH/$CHCl_3$ as eluents; or by preparative purification system *Büchi Sepacore*, consisting of a pump manager *C-615* with two pump modules *C-605* for binary solvent gradients, a *C-660* fraction collector, and *C-635* UV detector. Purification was carried out using an isocratic regime during the first 5 min at 5% of solvent B, and then linear gradient from 5% to 75% of solvent B for 30 min at a flow rate of 30 mL/min (A: water with 0.1% TFA, B: methanol with 0.1% TFA). Separations were made on prepacked preparative cartridges (150 × 40 mm) with reverse phase RP-18 silica gel (*Büchi* #54863).

Reverse phase HPLC purification was performed on an *Agilent 1100* series equipped with a binary pump system and a UV-visible detector using a *Phenomenex Luna* C18 100A (250 × 21.20 mm, 10 μm) preparative column. Purification was carried out using an isocratic regime during the first 5 min at 5% of solvent B and then linear gradients from 5% to 75% of solvent B for 30 min at a flow rate of 3 mL/min (A: water with 0.1% TFA, B: acetonitrile with 0.1% TFA). The fractions containing the products were freeze-dried, and their identity confirmed by ESI-MS and NMR.

NMR spectra were recorded on a *Varian Mercury 300*. Chemical shifts are given in ppm relative to TMS signal and coupling constants in Hz.

Time-gated emission measurements were made with a *Varian Cary Eclipse* Fluorescence Spectrophotometer. All measurements were made with a *Hellma* macro cuvette (111-QS) at 20 ºC, using the following settings: excitation wavelength 300 nm; excitation slit width 20.0 nm, emission slit width 10.0 nm; increment 1.0 nm; average time 0.2 s; total decay time 0.02 s; delay time 0.2 ms; PMT detector voltage 1000 V.

The steady state emission luminescence measurements were made in a *Jobin-Yvon Fluoromax-3* (*DataMax 2.20*), coupled to a temperature controller *Wavelength Electronics LFI-3751*. All measurements were performed at 20 ° C in a *Hellma* macro cuvette (111-QS), using the following parameters: excitation wavelength of 280 nm, bandwidth in the excitation slit 3.0 nm, bandwidth in the emission slit 6.0 nm, increase 1.0 nm, 0.2 s integration time. The emission spectrum was recorded between 295 and 500 nm.

EMSA was performed with a *BIO-RAS Mini Protean* gel system, powered by an electrophoresis power supplies *PowerPac Basic* model, maximum power 150 V, frequency 50.60 Hz at 140 V (constant V). Binding reactions were performed over 20 min at 8ºC in 20 mM Tris·HCl (pH 7.5), 90 mM KCl, 1.8 mM $MgCl_2$, 1.8 mM EDTA, 9% glycerol, 0.11 mg/mL BSA and 2.2 % NP-40. In the experiments we used 150 nM of the unlabeled *TAT* RNA. After incubation for 20 min products were resolved by PAGE using a 10% non-denaturing poliacrylamide gel and 0.5X TBE buffer for 45 min at 8 ºC, analyzed by staining with *SyBrGold* (Molecular Probes: 5 μL in 50 mL of 1X TBE) for 10 min, and visualized by fluorescence 5X TBE buffer (0.445 M Tris·HCl, 0.445 M Boric acid, 10 mM ETDA pH 8.0).

UV measurements were made in a *SmartSpec Plus* spectrophotometer using a disposable cuvette *trUView* from *BioRad*. Concentrations were measured using the listed extinction coefficients: 287,100 M^{-1} cm^{-1} at 260 nm for *TAR* RNA[1] and 5,579 M^{-1} cm^{-1} at 278 nm for Trp.[2]

Circular dichroism measurements were made with a *Jasco J-715* coupled to a *Neslab RTE-111* termostated water bath, using a *Hellma* macro cuvette (100-QS, 2 mm light pass). Measurements were made at 20 ºC. The spectra are the average of 4 scans.

General peptide synthesis procedures

Peptides were synthesized as C-terminal amides (usually in a 0.05 mmol scale using the resin Fmoc-PAL-PEG from *Life Technologies*: 0.25 mmol/g) on a *PS3 Peptide Synthesizer* (*Protein Technologies*) following standard Fmoc solid phase synthesis protocols. All peptide synthesis reagents and amino acid derivatives were purchased from *GL Biochem* (Shanghai) and *Novabiochem*; amino acids were purchased as protected Fmoc amino acids with the standard side chain protecting scheme: Fmoc-Pro-OH, Fmoc-Gly-OH, Fmoc-Thr(*t*Bu)-OH, Fmoc-Lys(Boc)-OH, Fmoc-Ile-OH and Fmoc-Arg(Pbf)-OH. All other chemicals were purchased from *Aldrich* or *Fluka*. All solvents were dry and synthesis grade.

Peptide bond-forming couplings were conducted for 40 min by using HBTU (10 equiv), DIEA (11 equiv) in DMF (2.5 mL) and 10 equivalents of the amino acids. Each amino acid was incubated for 2 min in the coupling mixture before being added onto the resin. After washing with DMF, the deprotection of the temporal Fmoc protecting group was performed by treating the resin with 20% piperidine in DMF solution for 15 min.

The final peptides were cleaved from the resin, and side-chain protecting groups were simultaneously removed using a standard TFA cleavage cocktail as outlined below. The High-Performance Liquid Chromatography (HPLC) was performed using an *Agilent 1100* series Liquid Chromatograph Mass Spectrometer system. Analytical HPLC was run using a *Zorbax Eclipse* XDB-C_{18} (5 µm) 4.6 × 150 mm analytical column from Agilent. The purification of the peptides was performed on a semipreparative *Jupiter Proteo 90A* (4 µm), 10 × 250 mm reverse-phase column from *Phenomenex*. The standard gradients used for analytical and preparative HPLC consisted on a linear gradient 5 → 95% CH_3CN, 0.1% TFA /H_2O, 0.1% TFA over 30 min, or on an isocratic regime during the first 5 min, followed by a linear gradient from 5% to 75% of solvent B for 30 min. Electrospray Ionization Mass Spectrometry (ESI/MS) was performed with an *Agilent 1100* Series LC/MSD VL G1956A model in positive scan mode using direct injection of the purified peptide solution into the MS.

Cleavage and deprotection of the resin bound peptides: The resin-bound peptide dried under argon (≈ 0.025 mmol) was placed in a 50 mL falcon tube and suspended in 2 mL of the cleavage cocktail (100 µL of CH_2Cl_2, 50 µL of water, 50 µL of triisopropylsilane (TIS) and TFA to 2 mL), and the resulting mixture was shaken for 3 h. The resin was filtered, and the TFA filtrate was concentrated under argon current to a volume of approximately 1 mL, which was then added to ice-cold diethyl ether (20 mL). After 10 min, the precipitate was centrifuged and washed again with 10 mL of ice-cold ether. The solid residue was dried under argon, redissolved in CH_3CN/H_2O 1:1 (1 mL) and purified by semipreparative reverse-phase HPLC. The collected fractions were lyophilized and stored at −20 °C.

---

1 The molar extinction coefficients of single strand oligos were calculated with the following formula: $\varepsilon_{260} = [(8.8 \times \#T) + (7.3 \times \#C) + (11.7 \times \#G) + (15.4 \times \#A)] \times 0.8 \cdot 103\ M^{-1}\ cm^{-1}$. Where, #T, #C, #G and #A are the number bases of each type in the sequence.

2 G.D. Fasman, *Handbook of Biochemistry and Molecular Biology, Proteins, I* **1976**, CRC Press, 3 ed., pp. 183–203.

1. Natural peptide **Tat**

1.1. Synthesis

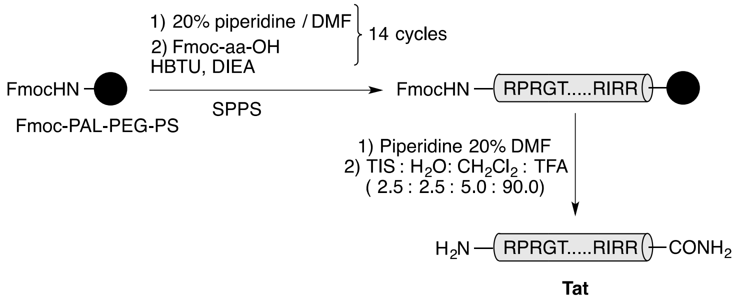

Scheme S1. Assembly of the peptide Tat in solid phase and subsequent cleavage/deprotection.

The peptide sequence was assembled in solid phase following the general solid-phase peptide synthesis methods, then deprotected and cleaved from the solid support using the standard TFA deprotection cocktail. Purification of the crude product by reverse-phase HPLC afforded **Tat** as a white solid in approximately 18% yield (8.7 mg).

Tat: (8.7 mg, aprox. 18% yield for a 0.025 mmol scale)
ESI-MS (m/z): $[MH]^+$ calcd. for $C_{69}H_{133}N_{37}O_{15}$= 1722.07, found: 1032.5 $[MH_2]^{2+}$ + 3 TFA, 650.4 $[MH_3]^{3+}$ + 2 TFA, 612.6 $[MH_3]^{3+}$ + 1 TFA, 574.4 $[MH_3]^{3+}$, 431.1 $[MH_4]^{4+}$, 345.0 $[MH_5]^{5+}$.
t_R = 11.3 min (*Eclipse XDB-C$_{18}$,* lineal gradient 5→95% CH_3CN, 0.1% TFA / H_2O, 0.1% TFA in 30 min).

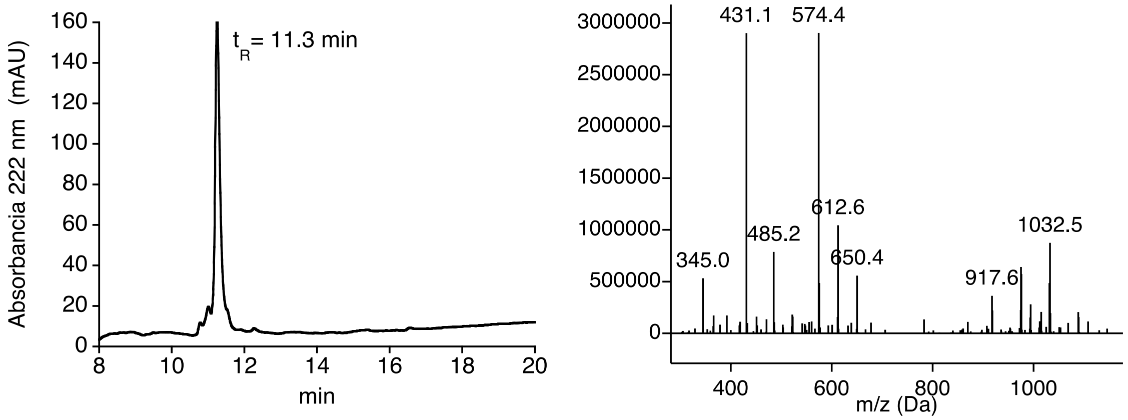

Figure S1. *Left*: HPLC trace of the reaction crude resulting from the cleavage of **Tat** peptide. *Right*: ESI-MS spectra corresponding to the peak at c.a. 11.3 min.

2. Peptide with a tryptophan antenna: **W-Tat-K(DOTA[Tb])**

2.1. Design and structure

Based on the solution structure of the peptide Tat bound to the BIV *TAR* hairpin RNA, as well as on mutational studies that identified the essential residues for high-affinity binding, we envisioned that extending the N-terminus of the Tat core sequence (Arg^{68} to Arg^{81}) with a Trp residue, and introducing a DOTA[Tb] complex at the C-terminus should result in a metallopeptide probe (**W-Tat-K(DOTA[Tb])** that, upon binding to its target *TAR* RNA, would fold into a β-hairpin and place both the DOTA[Tb] complex and the sensitizing indole side chain of the Trp close to each other. This might result in a significant increase in Tb(III) emission.

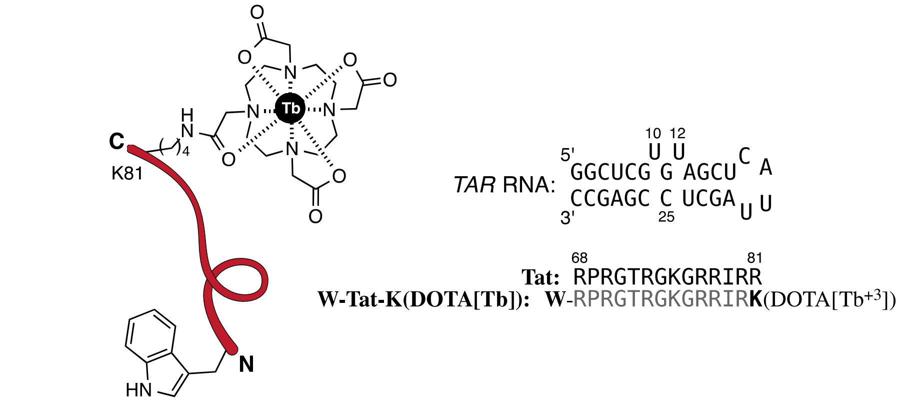

Figure S2. Sequences of the target BIV *TAR* RNA hairpin, natural BIV peptide **Tat** and the synthetic probe **W-Tat-K(DOTA[Tb])** are shown.

2.2. Synthesis

The target peptide **W-Tat-K(DOTA[Tb])** was assembled following standard Fmoc/tBu solid-phase peptide synthesis procedures; the DOTA chelating unit was introduced into the peptide scaffold as a tri-tBu-protected acid into the side chain of an orthogonally-deprotected Lys residue, while the peptide was still attached to the solid support. After purification the peptide was complexed with $TbCl_3$ in HEPES buffer to give the desired chelate, which was then repurified by reverse-phase HPLC (see below).

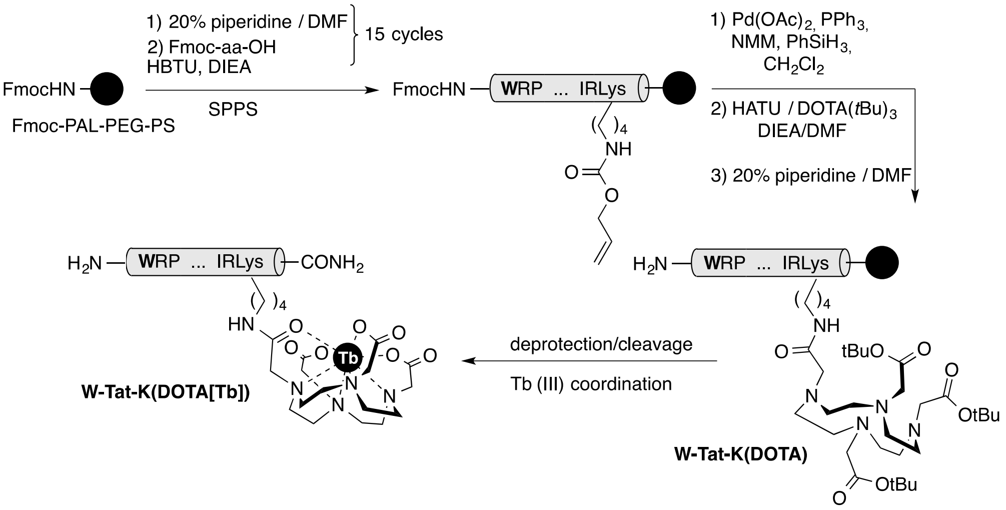

Scheme S2. Synthesis of the peptide with the DOTA unit orthogonally attached to the Lys side chain.

Deprotection of the orthogonally protected Lys(Alloc) side chain: Once the peptide was fully assembled in the solid phase, the side chain of the Lys(Alloc) residue is selectively deprotected for specific attachment of the DOTA unit. The detailed procedure is as follows: ≈ 0.05 mmol of peptide attached to the solid support were treated at room temperature for 12 h with a mixture of $Pd(OAc)_2$ (0.3 eq), PPh_3 (1.5 eq), *N*-Methylmorpholine (NMM, 10 eq), and $PhSiH_3$ (10 eq) in CH_2Cl_2 (2.5 mL). The resin was then filtered and washed with DMF (1 × 5 mL × 2 min), diethyldithiocarbamate (DEDTC) (25 mg in 5 mL of DMF, 2 × 5 min), DMF (2 × 5 mL × 2 min) and CH_2Cl_2 (2 × 5 mL × 2 min).

DOTA coupling to Lys side chain: DOTA(t-Bu)3 (46 mg, 0.08 mmol) was dissolved in dry DMF (470 µL). HATU (30 mg, 0.08 mmol) and *N,N*-diisopropylethylamine (DIEA) (21 µL, 0.12 mmol) were added to the solution. After two minutes, the resulting mixture was added over the Alloc-deprotected peptide attached to the resin (≈ 0.02 mmol). N2 was passed through the resin suspension for 6 h. The resin was

filtered, washed with DMF (3 × 3 mL × 3 min) and subjected to the final Fmoc deprotection step using standard conditions (20% piperidine/DMF).

Cleavage and deprotection of semipermanent protecting groups: The cleavage and deprotection of the resin-bound peptide was done following the experimental procedure already described. After reverse-phase HPLC purification the collected fractions were freeze-dried, and the desired peptide **W-Tat-K(DOTA)**, was isolated as white solid in a 16% yield.

W-Tat-K(DOTA): (4.4 mg, approx. 16% yield for a 0.02 mmol scale)
ESI-MS (m/z): $[MH]^+$ calcd. for $C_{96}H_{169}N_{41}O_{23}$= 2265.67, found 793.9 $[MH_3]^{3+}$ + 1 TFA, 756.0 $[MH_3]^{3+}$, 567.4 $[MH_4]^{4+}$, 453.9 $[MH_5]^{5+}$.
$\mathbf{t_R}$ = 12 min (*Eclipse XDB-C$_{18}$,* lineal gradient 5→95% CH_3CN, 0.1% TFA / H_2O, 0.1% TFA in 30 min).
UV (H_2O) λ_{max}: 278 nm; ε: 5,579 $M^{-1}cm^{-1}$.

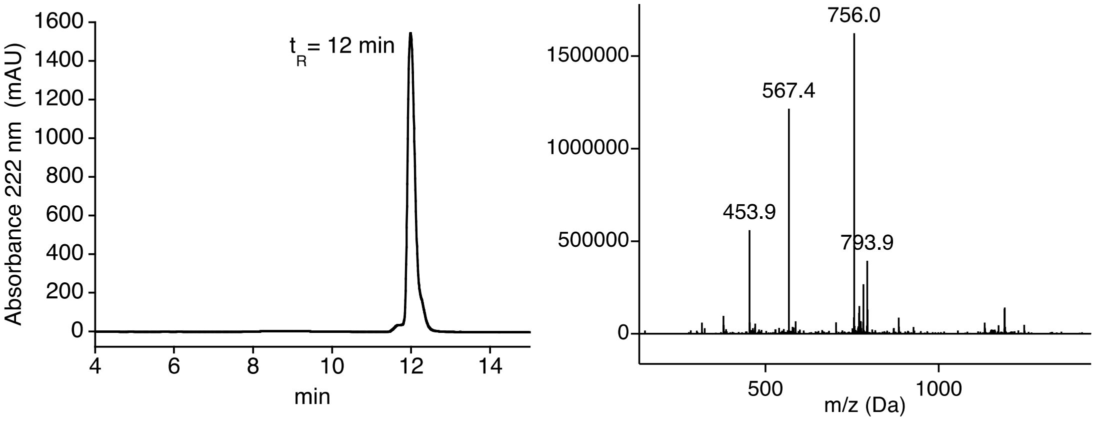

Figure S3. *Left*: HPLC trace of the pure product **W-Tat-K(DOTA)**. *Right*: ESI-MS spectra corresponding to the peak at c.a. 12 min.

Terbium chelation: The lyophilized peptide was dissolved in 200 µL HEPES buffer 50 mM, pH 7.6, NaCl 500 mM; and 300 µL of TbCl3 50 mM solution in HCl 1 mM was added. Then, the solution was brought of 1 mL in water. The mixture was shaken for 6 h and then HPLC-purified. After lyophilization, a white solid was obtained and identified as the desired terbium-peptide complex.

W-Tat-K(DOTA[Tb]): (1.8 mg, 73%)
ESI-MS (m/z): $[MH]^+$ calcd. for $C_{96}H_{166}N_{41}O_{23}Tb$= 2421.57, found 1325.6 $[MH_2]^{2+}$ + 2 TFA, 883.8 $[MH_3]^{3+}$ + 2 TFA, 845.7 $[MH_3]^{3+}$ + 1 TFA, 807.9 $[MH_3]^{3+}$, 634.6 $[MH_4]^{4+}$ + 1 TFA, 606.2 $[MH_4]^{4+}$, 485.2 $[MH_5]^{5+}$.
$\mathbf{t_R}$ = 12.3 min (column *Eclipse XDB-C$_{18}$,* lineal gradient 5→95% CH_3CN, 0.1% TFA / H_2O, 0.1% TFA in 30 min).
UV (H_2O) λ_{max}: 278 nm; ε: 5,579 $M^{-1}cm^{-1}$.

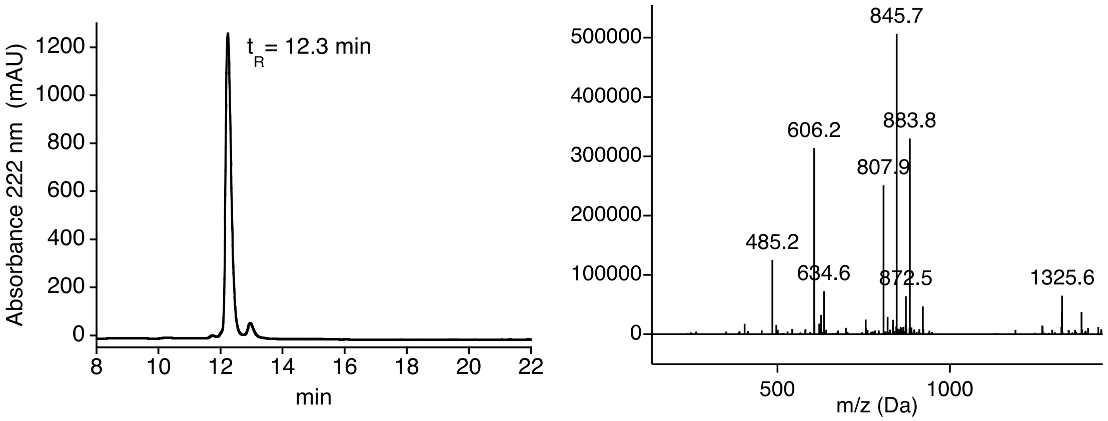

Figure S4. *Left*: HPLC trace of the pure lanthanide complex **W-Tat-K(DOTA[Tb])** peptide. *Right*: ESI-MS spectra corresponding to the peak at c.a. 12.3 min.

2.3. Biophysical assays

Luminescence measurements and EMSA experiments

Having at hand the desired metallopeptide probe, we studied the effect of *TAR* RNA in its emission. As expected, the peptide **W-Tat-K(DOTA[Tb])** was weakly emissive by itself. Unfortunately, addition of the target *TAR* BIV RNA did not promote any increase in its luminescence (Figure S5a). Control electrophoretic mobility assays (EMSA) in polyacrylamide gel under non-denaturing conditions showed that the peptide was binding to the RNA. Thus, incubation of the BIV *TAR* hairpin oligonucleotide with increasing concentrations of the peptide **W-Tat-K(DOTA[Tb])** gave rise to new slow-migrating bands, consistent with the formation of the expected peptide/RNA complex (Figure S5b, band 2). Given that the probe appears to maintain the RNA binding capabilities of the parent Tat peptide (Figure S5b, band 1), the lack of fluorescent emission suggests that the antenna and the Tb complex are too distant for an efficient transfer. Alternatively, the failing might arise from the rapid quenching of the excited state of the Trp by the RNA bases before the energy is transferred to the Tb(III) complex. This is consistent with reports of Trp quenching due to oligonucletoide binding, as well as on the quenching observed in the fluorescence emission of the Trp residue in our own system upon excitation at 300 nm (Figure S5c).

Time-gated emission of peptide W-Tat-K(DOTA[Tb]) with *TAR* RNA (Figure S5a). To 3 mL of a 100 nM solution of the peptide **W-Tat-K(DOTA[Tb])** in HEPES buffer (10 mM HEPES pH 7.6, 100 mM NaCl), 1 equivalent of *TAR* RNA was added. The emission spectra were recorded before and after the addition.

Binding assays of Tat and W-Tat-K(DOTA[Tb]) with *TAR* RNA (Figure S5b): Increasing concentrations of the natural peptide **Tat**, and the peptide **W-Tat-K(DOTA[Tb])** were incubated with the target RNA *TAT* (5'-GGC UCG UGU AGC UCA UUA GCU CCG AGC C-3') for 20 min and analysed by EMSA.

Steady-state emission of peptide W-Tat-K(DOTA[Tb]) with *TAR* RNA (Figure S5c). To 3 mL of a 100 nM solution of the peptide **W-Tat-K(DOTA[Tb])** in HEPES buffer (10 mM HEPES pH 7.6, 100 mM NaCl), 1 equivalent of *TAR* RNA was added. The emission spectra were recorded before and after the addition.

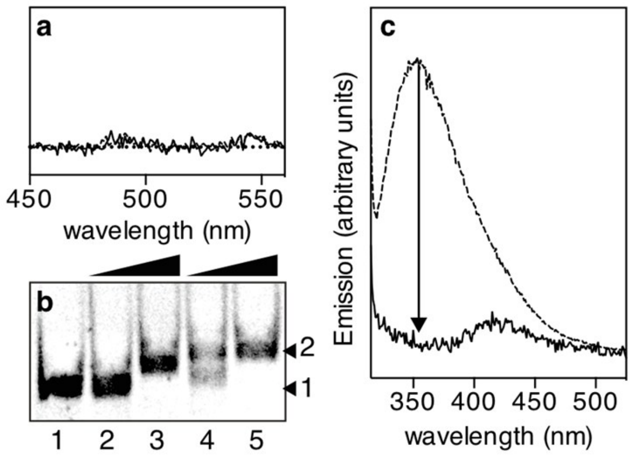

Figure S5. a) Time-resolved luminescence of the Tb(III) ion in **W-Tat-K(DOTA[Tb])** in absence (dashed lines) and in the presence of the target *TAR* RNA hairpin (solid lines); b) Binding of **W-Tat-K(DOTA[Tb])** to the *TAR* RNA by EMSA. Lanes 2 and 3: 100, 500 nM of **Tat**; lanes 4, 5: 100 and 500 nM of **W-Tat-K(DOTA[Tb])**. All EMSA lanes with 150 nM *TAR* RNA in 20 mM Tris·HCl pH 7.5, 90 mM KCl, 1.8 mM $MgCl_2$, 1.8 mM edta, 9% glycerol, 0.11 mg/mL BSA, 2.2% NP-40; c) Fluorescence emission of **W-Tat-K(DOTA [Tb])** in absence (dashed lines) and in the presence of the target *TAR* RNA (solid lines).

3. Alternative peptides

3.1. Design and structures

Despite the failing of the probe, the good RNA binding affinity of this first design encouraged us to explore other alternatives. Therefore, we tried adding spacers, such as 5-amino-3-oxapentanoic acid (**O1Pen**), or polyprolyne helices (**P$_4$**) between the Trp antenna and the peptide binding module, as well as switching the position of the antenna and the DOTA[Tb] unit. We also synthesized probes containing other antennas than Trp, such as 7-methoxycoumarin (**Cou**), thiazole orange (**TO**), or acridine (**ACR**), which are known to increase their emission upon binding to the RNA (see Table 1, below). Unfortunately, none of those new probes lighted up in the presence of the target *TAR* RNA hairpin.

Table 1. Peptide probes used in this study. **Cou** = 7-methoxycoumarin, O1Pen = 5-amino-3-oxapentanoic acid, **ACR** = acridine, and **TO** = thiazole orange; **ϕ** = 1,10-phenanthroline-5-carbonyl group.

**Tat**	RPRGTRGKGRRIRR
**W-Tat-K(DOTA[Tb])**	W-RPRGTRGKGRRIRK**(DOTA[Tb])**
**EDTA[Tb]-Tat-Lys(ϕ)**	**EDTA**-RPRGTRGKGRRIRK(ϕ)
**(DOTA[Tb])-Tat-W**	**(DOTA[Tb])**-RPRGTRGKGRRIR-W
**W-O1Pen-Tat-K(DOTA[Tb])**	W-O1Pen-RPRGTRGKGRRIRK**(DOTA[Tb])**
**W-G-Tat-K(DOTA[Tb])**	W-G-RPRGTRGKGRRIRK**(DOTA[Tb])**
**W-P-Tat-K(DOTA[Tb])**	W-P-RPRGTRGKGRRIRK**(DOTA[Tb])**
**W-W-Tat-K(DOTA[Tb])**	W-W-RPRGTRGKGRRIRK**(DOTA[Tb])**
**Cou-Tat-K(DOTA[Eu])**	**Cou**-RPRGTRGKGRRIRK**(DOTA[Eu])**
**Cou-P$_4$-Tat-P$_4$-K(DOTA[Eu])**	**Cou**-PPPP-RPRGTRGKGRRIR-PPPPK**(DOTA[Eu])**
**ACR-Tat-K(DOTA[Eu])**	**ACR**-RPRGTRGKGRRIRK**(DOTA[Eu])**
**TO-Tat-K(DOTA[Eu])**	**TO**-RPRGTRGKGRRIRK**(DOTA[Eu])**

3.2. Synthesis of small molecular fragments and assembly of peptides

*Synthesis of the acridine derivative **1***

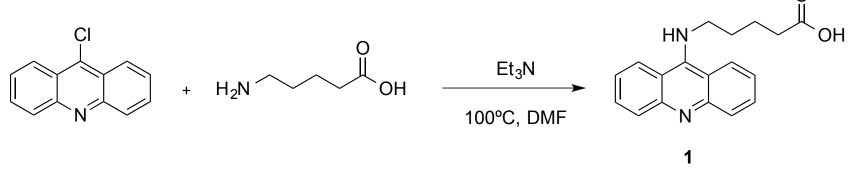

9-chloroacridine (150 mg, 0.7 mmol), 5-aminovaleric acid (91 mg, 0.77 mmol) and triethylamine (72 mg, 1.54 mmol) were added to a round bottom flask and dissolved in ~7 mL of DMF. The reaction mixture was refluxed at 100 deg under Ar overnight. The solvents were removed under reduced pressure, and the resulting residue was purified by preparative reverse-phase chromatography. The combined fractions were concentrated and freeze-dried yielding a yellow oil that was identified as the desired product (**1**, 147 mg, 71%).

^{1}H NMR δ (300 MHz, MeOH-*d$_4$*): 8.49 (d, *J* = 8.6 Hz, 2H), 7.95 (t, *J* = 7.2 Hz, 2H), 7.79 (d, *J* = 8.5 Hz, 2H), 7.56 (t, *J* = 7.3 Hz, 2H), 4.16 (t, *J* = 7.2 Hz, 2H), 2.42 (t, *J* = 7.0 Hz, 2H), 2.03 (q, *J* = 7.6 Hz, 2H), 1.78 (q, *J* = 7.1 Hz, 2H).
^{13}C NMR δ (MeOH-*d$_4$*): 177.2 (C), 159.7 (C), 141.3 (C), 136.5 (CH), 124.9 (CH), 119.6 (CH), 114.0 (C), 50.2 (CH_2), 45.7 (CH_2), 30.0 (CH_2), 23.1 (CH_2).
t_R = 16.1 min (*Eclipse XDB-C$_{18}$,* lineal gradient 5→95% CH_3CN, 0.1% TFA / H_2O, 0.1% TFA in30 min).
HR-ESI-MS (m/z): $[MH]^+$ calc. for $C_{18}H_{18}N_2O_2$ = 295.1441, found $[MH]^+$ 295.1447.

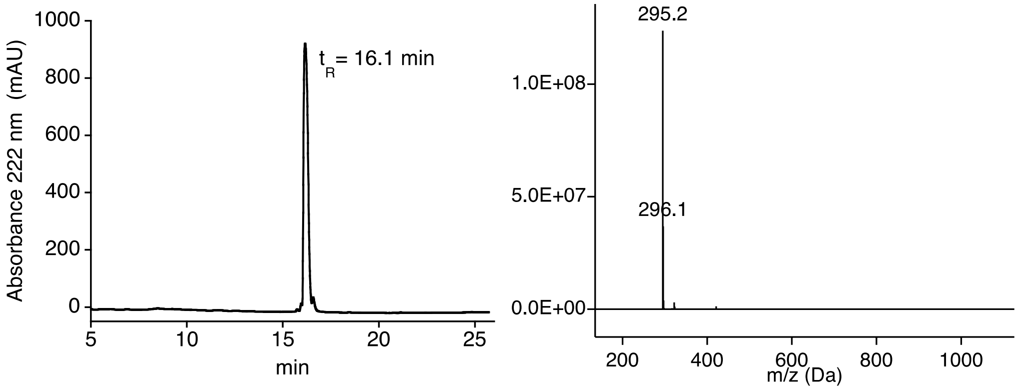

Figure S6. *Left*: HPLC trace of the purified acridine (**1**) (222 nm). *Right*: ESI-MS of **1** (m/z): $[MH]^+$ calculated for $C_{18}H_{18}N_2O_{21}$ = 295.14, found 295.2. UV (H_2O) λ_{max}, nm (ε): 434 (7624) $M^{-1}cm^{-1}$.

The molar extinction coefficient of compound **1** was obtained by linear regression analysis of the UV absorption values of samples of known concentrations by weight. ε_{434nm} = 7624 $M^{-1}cm^{-1}$ was found for the acridine derivative **1**.

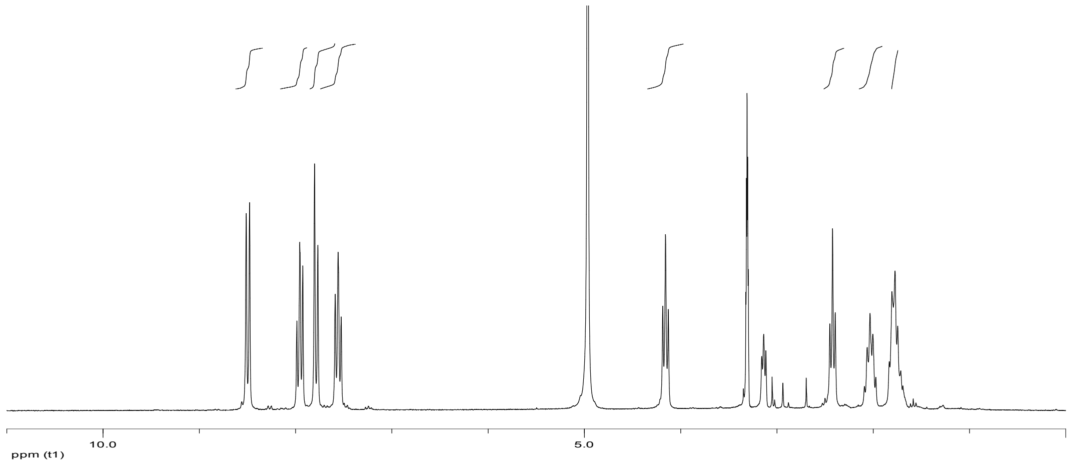
10.0
5.0
ppm (t1)

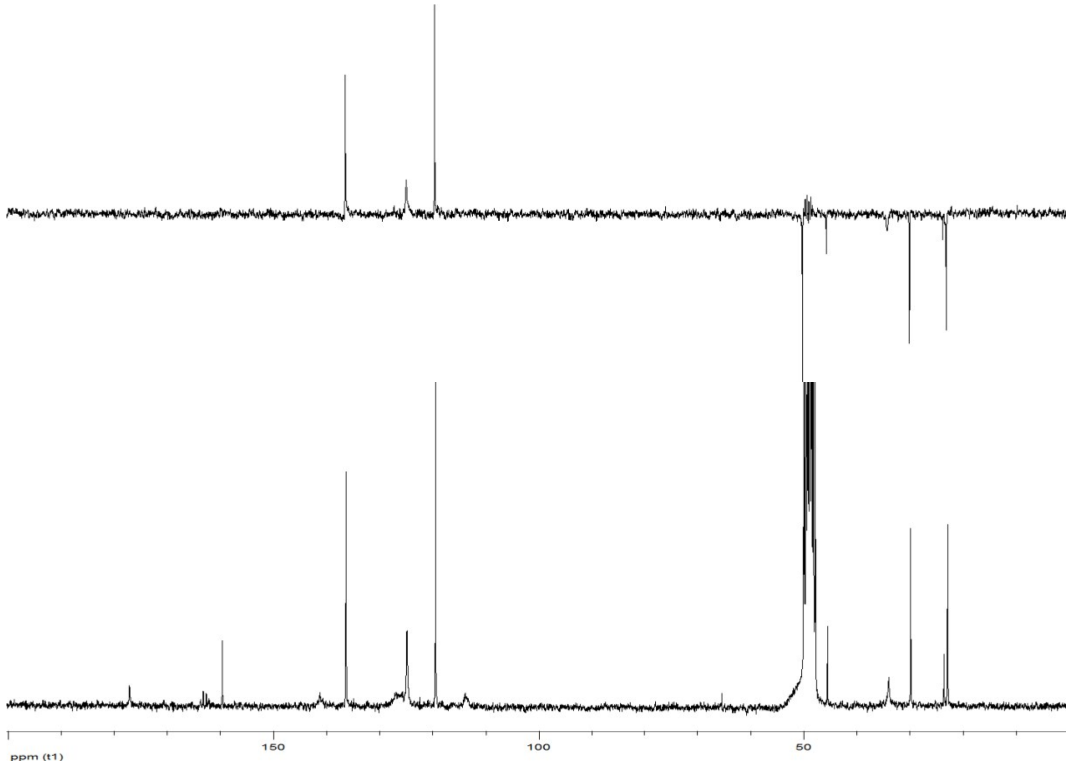
150
100
50
ppm (t1)

*Synthesis of 1-(carboxymethyl)- 4-methyl-chinolinium bromide (**2**)*

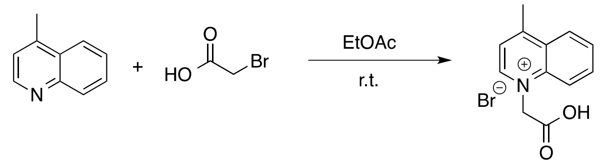

2

Compound **2** was synthesized following previously described procedures.[3] 4-methylquinoline was added (6.2 g, 43 mmol) to a bromoacetic acid solution (6.1 g, 44 mmol) in ethyl acetate (~3 mL). The reaction mixture was stirred at rt overnight. The product appeared as a pale yellow precipitate, which was filtered and washed with EtOAc. The residue was adsorbed in silica and purified by flash column chromatography (20% MeOH/$CHCl_3$) to give a solid identified as the desired product (3.4 g, 35%).

^{1}H NMR δ (300 MHz, MeOH-*d_4*): 9.24 (d, *J* = 6.1 Hz, 1H), 8.61 (d, *J* = 8.5 Hz, 1H), 8.34 (d, *J* = 9.0 Hz, 1H), 8.26 (m, 1H), 8.1 (m, 2H), 5.95 (s, 2H), 3.12 (s, 3H).
ESI-MS (m/z): $[MH]^+$ calc. for $C_{12}H_{12}NO_2$ = 202.2, found $[MH]^+$ 202.0.

*Synthesis of thiobenzoxazole (**3**)*

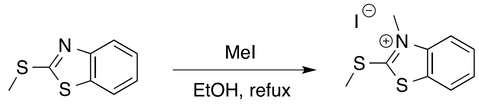

3

The benzothiazole derivative was synthesized following reported procedures.[3] Iodomethane (4.1 mL, 67 mmol) was added to a solution of 2-(methylthio)benzothiazole (6.0 g, 33 mmol) in EtOH (15 mL). The mixture was refluxed overnight affording a yellow precipitate, which was filtered and washed with cold EtOH. The resulting light-yellow solid was identified as the desired product (**3**, 2.8 g, 16% yield).

^{1}H NMR δ (300 MHz, MeOH-*d4*): 8.25 (d, *J* = 8.26 Hz, 1H), 8.10 (d, *J* = 8.10 Hz,1H), 7.87 (ddd, *J* = 8.5, 7.4, 1.2 Hz, 1H), 7.74 (ddd, *J* = 8.3, 7.4, 1.0 Hz, 1H), 4.18 (s, 3H), 3.16 (s, 3H).
ESI-MS (m/z): $[MH]^+$ calc. for $C_9H_{11}NS_2$ = 196.3, found $[MH]^+$ 196.5.

*Synthesis of thiazole orange derivative **4***

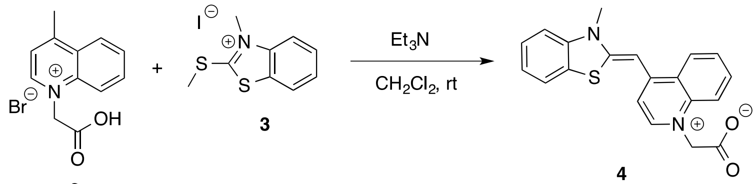

The synthesis of the thiazole orange derivative was performed as previously described.[4] To a solution of benzothiazole derivative **3** (200 mg, 0.9 mmol) and the quinoline **2** (140 mg, 0.7 mmol) in 14 mL of CH_2Cl_2, triethylamine (251 µL, 1.8 mmol) was added. The mixture was stirred in the dark at rt for 17 h. The solvent was evaporated under reduced pressure, the residue was redissolved in 30 mL of hot MeOH, and added over a round bottom flask containing 75 mL of H_2O. The resulting solution was stored at 4 ºC

3 Y. Hara, T. Fujii, H. Kashida, K. Sekiguchi, X. Liang, K. Niwa, T. Takase, Y. Yoshida, H. Asanuma, *Angew. Chemie Int. Ed.*, 2010, **49**, 5502–5506.

4 L. Bethge, D. V. Jarikote, O. Seitz, *Bioorg. Med. Chem.*, **2008**, *16*, 114–125.

for 4 days to afford a precipitate, which was collected by filtration, washed with hexane and cold water, and freeze-dried to yield a red solid that was identified as the desired product (**4**, 73 mg, 30% yield).

^{1}H NMR δ (300 MHz, DMSO-d_6): 8.77 (d, J = 8.5 Hz, 1H), 8.50 (d, J = 7.4 Hz, 1H), 8.10 (d, J = 7.9 Hz, 1H), 7.92 (t, J = 7.9 Hz, 1H), 7.82 (t, J = 7.8 Hz, 2H), 7.70 (t, J = 7.6 Hz, 1H), 7.62 (t, J = 7.8 Hz, 1H), 7.41 (m, 2H), 6.96 (s, 1H), 5.46 (s, 2H), 4.04 (s, 3H).

ESI-MS (m/z): $[MH]^+$ calculated for $C_{20}H_{16}N_2O_2S$ = 348.4, found 349.1 $[MH]^+$.

*Synthesis of peptides **W-O1Pen-Tat-K(DOTA [Tb])**, **W-G-Tat-K(DOTA [Tb])**, **W-P-Tat-K(DOTA [Tb])** and **W-W-Tat-K(DOTA [Tb])***

The synthetic route and experimental procedure for the synthesis of peptides **W-O1Pen-Tat-K(DOTA [Tb])**, **W-G-Tat-K(DOTA [Tb])**, **W-P-Tat-K(DOTA [Tb])** and **W-W-Tat-K(DOTA [Tb])** was the same than the previously described for peptide **W-Tat-K(DOTA [Tb])**.

W-O1Pen-Tat-K(DOTA[Tb]): (10 mg, aprox. 16% yield for 0.02 mmol scale)
ESI-MS (m/z): $[MH]^+$ calcd. for $C_{99}H_{170}N_{41}O_{25}Tb$= 2493.64, found 1261.8 $[MH_2]^{2+}$, 803.1 $[MH_4]^{4+}$ + 6 TFA.
t_R = 12.6 min (*Eclipse XDB-C$_{18}$,* lineal gradient 5→95% CH_3CN, 0.1% TFA / H_2O, 0.1% TFA in 30 min).
UV (H_2O) λ_{max}: 278 nm; ε: 5,579 $M^{-1}cm^{-1}$.

W-G-Tat-K(DOTA[Tb]): (1.5 mg, aprox. 5% yield for 0.02 mmol scale)
ESI-MS (m/z): $[MH]^+$ calcd. for $C_{98}H_{168}N_{41}O_{25}Tb$= 2479.6, found 1297.3 $[MH_2]^{2+}$ + 1 TFA, 903.4 $[MH_3]^{3+}$ + 2 TFA, 865.0 $[MH_3]^{3+}$ + 1 TFA, 826.8 $[MH_3]^{3+}$, 649.1 $[MH_4]^{4+}$ + 1 TFA, 620.3 $[MH_4]^{4+}$.
t_R = 12.6 min (*Eclipse XDB-C$_{18}$,* lineal gradient 5→95% CH_3CN, 0.1% TFA / H_2O, 0.1% TFA in 30 min).
UV (H_2O) λ_{max}: 278 nm; ε: 5,579 $M^{-1}cm^{-1}$.

W-P-Tat-K(DOTA[Tb]): (2.5 mg, aprox. 8% yield for 0.02 mmol scale)
ESI-MS (m/z): $[MH]^+$ calcd. for $C_{101}H_{172}N_{41}O_{25}Tb$= 2519.7, found 916.3 $[MH_3]^{3+}$ + 2 TFA, 878.9 $[MH_3]^{3+}$ + 1 TFA, 840.3 $[MH_3]^{3+}$, 630.6 $[MH_4]^{4+}$.
t_R = 13.4 min (*Eclipse XDB-C$_{18}$,* lineal gradient 5→95% CH_3CN, 0.1% TFA / H_2O, 0.1% TFA in 30 min).
UV (H_2O) λ_{max}: 278 nm; ε: 5,579 $M^{-1}cm^{-1}$.

W-W-Tat-K(DOTA[Tb]): (3.8 mg, aprox 9% yield for 0.02 mmol scale)
ESI-MS (m/z): $[MH]^+$ calcd. for $C_{107}H_{175}N_{42}O_{25}Tb$= 2608.8, found 945.8 $[MH_3]^{3+}$ + 2 TFA, 908.1 $[MH_3]^{3+}$ + 1 TFA, 869.9 $[MH_3]^{3+}$, 681.9 $[MH_4]^{4+}$ + 1 TFA.
t_R = 13.4 min (*Eclipse XDB-C$_{18}$,* lineal gradient 5→95% CH_3CN, 0.1% TFA / H_2O, 0.1% TFA in 30 min).
UV (H_2O) λ_{max}: 278 nm; ε: 5,579 $M^{-1}cm^{-1}$.

*Synthesis of peptide **(DOTA [Tb])-Tat-W***

The strategy for the synthesis of peptide **(DOTA [Tb])-Tat-W** consisted on the coupling of the DOTA unit (following the already described procedure) to the N-terminal end of the peptide after assembling the peptide chain by SPPS. After RP-HPLC purification, the terbium coordination proceeded as previously described.

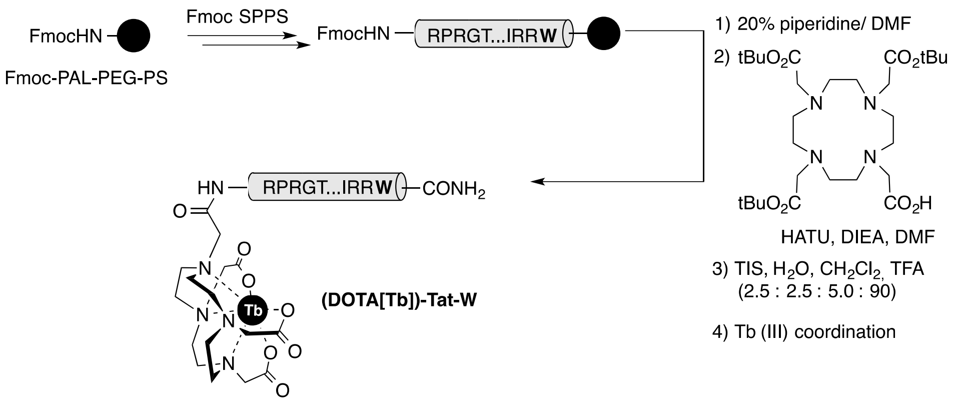

Scheme S3. Synthetic route for the synthesis of **(DOTA[Tb])-Tat-W**.

(DOTA[Tb])-Tat-W: (5.3 mg, aprox. 10% yield for 0.02 mmol scale)
ESI-MS (m/z): $[MH]^+$ calcd. for $C_{90}H_{154}N_{39}O_{22}Tb$ = 2294.4, found 841.1 $[MH_3]^{3+}$ + 2 TFA, 803.0 $[MH_3]^{3+}$ + 1 TFA, 765.6 $[MH_3]^{3+}$, 574.0 $[MH_4]^{4+}$.
t_R **=** 13 min (*Eclipse XDB-C₁₈,* lineal gradient 5→95% CH_3CN, 0.1% TFA / H_2O, 0.1% TFA in 30 min).
UV (H_2O) λ_{max}: 278 nm; ε: 5,579 $M^{-1}cm^{-1}$.

*Synthesis of peptides **Cou-Tat-K(DOTA[Eu]), Cou-P₄-Tat-P₄-K(DOTA [Eu]), ACR-Tat-K(DOTA [Eu]) and TO-Tat-K(DOTA [Eu])***

The synthetic tactic for the obtention of peptides **Cou-Tat-K(DOTA[Eu])**, **Cou-P_4-Tat-P_4-K(DOTA [Eu])**, **ACR-Tat-K(DOTA [Eu])** and **TO-Tat-K(DOTA [Eu])** is shown is the scheme below.

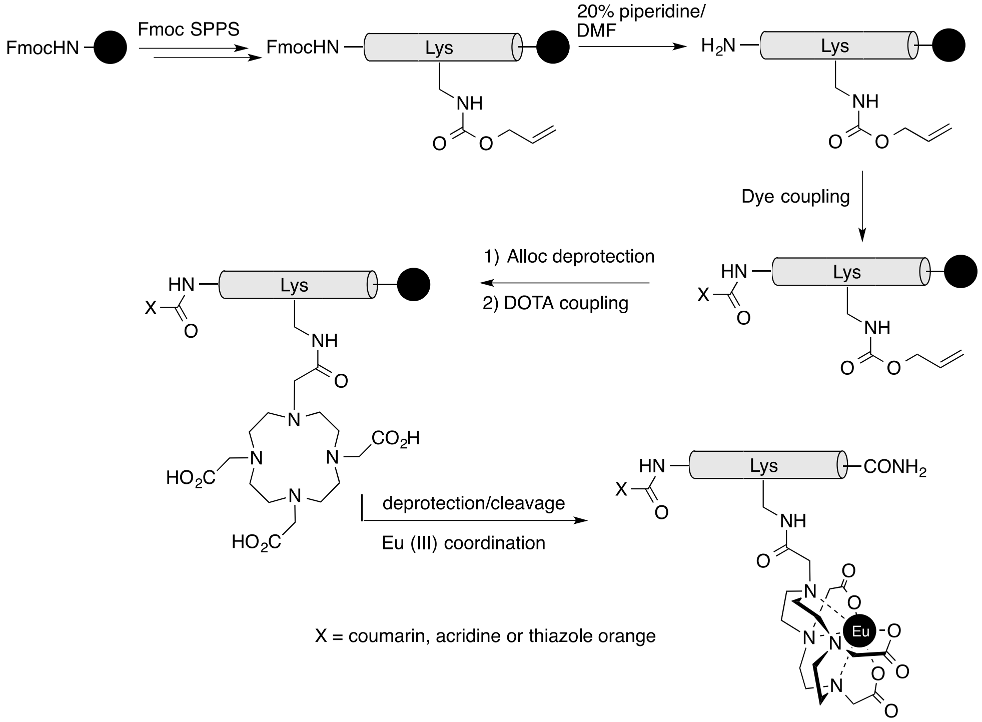

Scheme S4. Syntehic route for the obtention of peptides **Cou-Tat-K(DOTA[Eu])**, **Cou-P_4-Tat-P_4-K(DOTA [Eu])**, **ACR-Tat-K(DOTA [Eu])** and **TO-Tat-K(DOTA [Eu])**.

Peptide chain elongation and Fmoc deprotection: The peptide chains of each of the four peptides were synthesized automatically in solid phase using the resin PAL-PEG-PS as solid support and in a 0.05 mmol

scale, following the general protocol for solid phase synthesis already described. Once the peptides were fully assembled in the solid phase, the N-terminal temporary protecting group was deprotected using the standard conditions (20% piperidine/ DMF) for the subsequent coupling of coumarine, thiazole orange or acridine derivatives over the liberated amines.

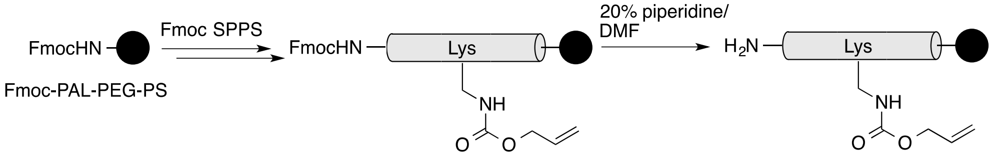

Scheme S5. SPPS and Fmoc deprotection for the subsequent coupling of the corresponding dyes.

Coupling of 7-methoxycoumarin-3-carboxilic acid: The commercially available 7-methoxycoumarin-3-carboxilic acid (11 mg, 0.05 mmol) and HATU (19 mg, 0.05 mmol) were dissolved in 250 μL of DMF. Then, 9.4 μL of DIEA (0.55 mmol) were added to the solution and after activating the mixture for 2 minutes, the solution was added over the peptide resins (≈ 0.025 mmol). The mixtures were shaken for two hours and then the resins were filtered and washed with DMF (3 × 3 mL × 3 min).

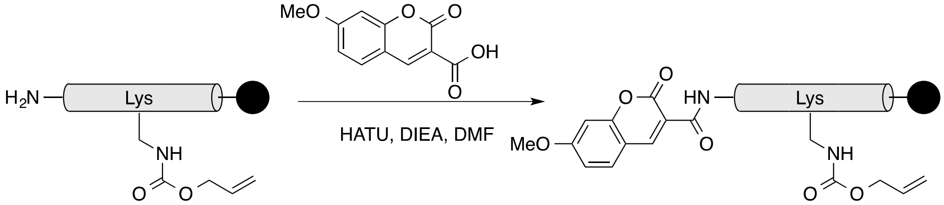

Scheme S6. Attachement of the coumarin unit to the N-terminal end of the peptide chains.

Coupling of acridine derivative (1): 1 (42.7 mg, 0.2 mmol) and HATU (76 mg, 0.2 mmol) were dissolved in DMF (667 μL) and then DIEA (28.43 mg, 0.22 mmol, 38 μL) was added to the solution. After 2 min, the resulting mixture was added over the amino peptide attached to the resin (≈ 0.025 mmol) and N_2 was passed through the resin suspension for 3 h. Then, the resin was filtered and washed with DMF (2 × 5 mL × 3 min).

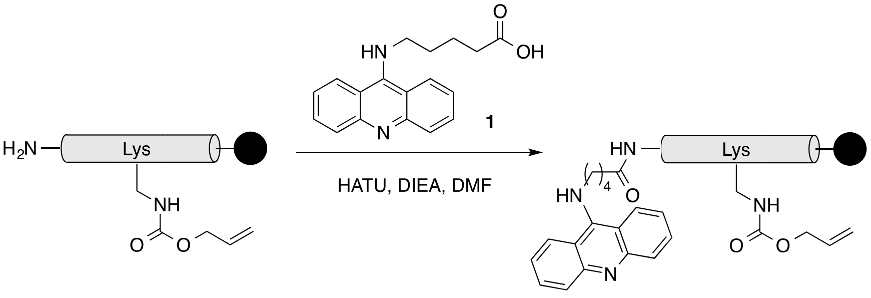

Scheme S7. Coupling of the acridine derivative **1** to the N-terminal end of the peptide.

Coupling of thiazole orange derivative (4): Thiazole orange (**4**) (8.7 mg, 0.025 mmol), PyAOP (13 mg, 0.025 mmol), N-methylmorpholine (NMM, 3 μL, 0.027 mmol,) and pyridinium *p*-toluensulfonate (PPTS, 6.3 mg, 0.025 mmol) were dissolved in 275 μL of anhydrous DMF. The mixture was actived for 2 minutes and then it was added over a mixture of the amine resin (≈ 0.025 mmol) and NMM (3 μL, 0.027 mmol,) in 227 μL of anhydrous DMF. N_2 was passed through the resin suspension for 3 h. Then, the resin was filtered and washed with DMF (2 × 5 mL × 3 min) and CH_2Cl_2 (2 × 5 mL × 3 min).

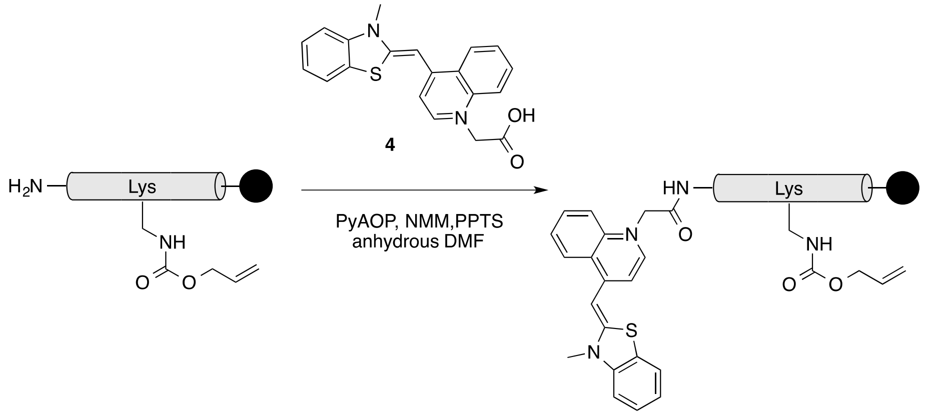

Scheme S8. Coupling of **4** to the N-terminal amine of the resin-bound peptide.

Alloc deprotection and DOTA tris (*t*-Bu) ester coupling: Once that each of the dyes were coupled to the N-terminal end of the respective peptides, the side chain of the Lys(Alloc) residue is selectively deprotected for specific attachment of the DOTA unit. The experimental procedures for the deprotection of the orthogonally protected Lys(Alloc) side chain and DOTA coupling to Lys side chain, were the same than the previously described.

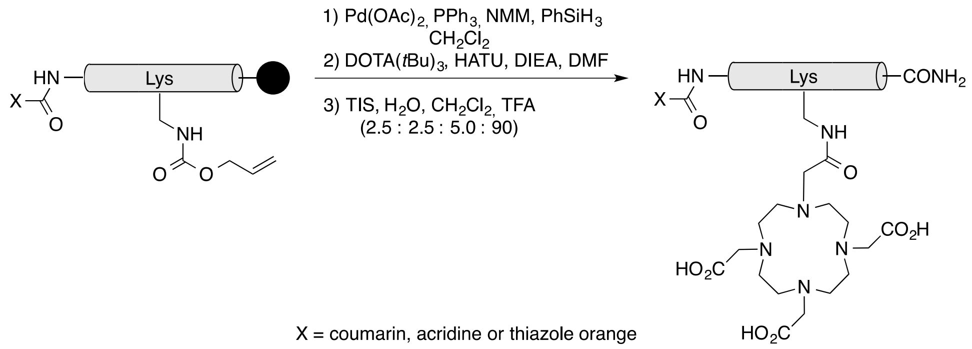

Scheme S9. Alloc deprotection and DOTA coupling following the already described procedures.

Eu^{3+} coordination: The lyophilized peptides were dissolved in 200 μL HEPES buffer 50 mM, pH 7.6, NaCl 500 mM; and 300 μL of $EuCl_3$ 50 mM solution in HCl 1 mM were added. Then, the solutions were brought to 1 mL in water. The mixtures were shaken for 6 h and after checking by HPLC-MS that the coordination had occurred they were HPLC-purified. After lyophilization, a white solids were obtained and identified as the desired europium-peptide complexes, **Cou-Tat-K(DOTA[Eu])**, **Cou-P_4-Tat-P_4-K(DOTA[Eu])**, **ACR-Tat-K(DOTA[Eu])** and **TO-Tat-K(DOTA[Eu])**.

Cou-Tat-K(DOTA[EU]): (1.9 mg, aprox. 50.8% yield for a 0.025 mmol scale)

ESI-MS (m/z): $[MH]^+$ calcd. for $C_{96}H_{161}EuN_{38}O_{27}$= 2431.6, found 1330.0 $[MH_2]^{2+}$ + 2 TFA, 886.8 $[MH_3]^{3+}$ + 2 TFA, 848.8 $[MH_3]^{3+}$ + 1 TFA, 810.7 $[MH_3]^{3+}$, 636.8 $[MH_4]^{4+}$ + 1 TFA, 608.4 $[MH_4]^{4+}$, 487.2 $[MH_5]^{5+}$, 406.0 $[MH_6]^{6+}$.
t_R = 13.2 min (*Eclipse XDB-C_{18},* lineal gradient 5→95% CH_3CN, 0.1% TFA / H_2O, 0.1% TFA in 30 min).
UV (H_2O) λ_{max}: 350 nm; ε: 26,000 $M^{-1}cm^{-1}$.

Cou-P_4-Tat-P_4-K(DOTA[EU]): (2.3 mg, aprox. 69.2% yield for a 0.025 mmol scale)
ESI-MS (m/z): $[MH]^+$ calcd. for $C_{139}H_{224}EuN_{51}O_{35}$= 3321.6, found 1222.3 $[MH_3]^{3+}$ + 3 TFA, 916.2 $[MH_4]^{4+}$ + 3 TFA, 888.3 $[MH_4]^{4+}$ + 2 TFA, 859.5 $[MH_4]^{4+}$ + 1 TFA.
t_R = 14.1 min (*Eclipse XDB-C_{18},* lineal gradient 5→95% CH_3CN, 0.1% TFA / H_2O, 0.1% TFA in 30 min).
UV (H_2O) λ_{max}: 350 nm; ε: 26,000 $M^{-1}cm^{-1}$.

ACR-Tat-K(DOTA[EU]): (2.1 mg, aprox. 69.9% yield for a 0.025 mmol scale)
ESI-MS (m/z): $[MH]^+$ calcd. for $C_{103}H_{172}EuN_{41}O_{23}$ = 2504.74, found 950.7 $[MH_3]^{3+}$ + 3 TFA, 911.6 $[MH_3]^{3+}$ + 2 TFA, 873.2 $[MH_3]^{3+}$ + 1 TFA, 655.4 $[MH_4]^{4+}$ + 1 TFA, 502.1 $[MH_5]^{5+}$
$\mathbf{t_R}$ = 13.5 min (*Eclipse XDB-C$_{18}$,* lineal gradient 5→95% CH_3CN, 0.1% TFA / H_2O, 0.1% TFA in 30 min).
UV (H_2O) λ_{max}: 434 nm; ε: 7,624 $M^{-1}cm^{-1}$.

TO-Tat-K(DOTA[EU]): (0.5 mg, aprox. 78.1% yield for a 0.025 mmol scale)
ESI-MS (m/z): $[MH]^+$ calcd. for $C_{105}H_{172}EuN_{41}O_{23}S$= 2559.8, found 967.8 $[MH_3]^{3+}$ + 3 TFA, 929.3 $[MH_3]^{3+}$ + 2 TFA, 892.0 $[MH_3]^{3+}$ + 1 TFA, 698.3 $[MH_4]^{4+}$ + 2 TFA, 640.6 $[MH_4]^{4+}$, 513.1 $[MH_5]^{5+}$,
$\mathbf{t_R}$ = 14.9 min (*Eclipse XDB-C$_{18}$,* lineal gradient 5→95% CH_3CN, 0.1% TFA / H_2O, 0.1% TFA in 30 min).
UV (H_2O) λ_{max}: 510 nm; ε: 65,000 $M^{-1}cm^{-1}$.

3.3. Biophysical assays

Luminescence measurements

Time-gated and steady state experiments with peptide W-O1Pen-Tat-K(DOTA [Tb]): To 3 mL of a 100 nM solution of the peptide **W-O1Pen-Tat-K(DOTA [Tb])** in HEPES buffer (10 mM HEPES pH 7.6, 100 mM NaCl), 1 equivalent of *TAR* RNA was added. The emission spectra were recorded before and after each addition.

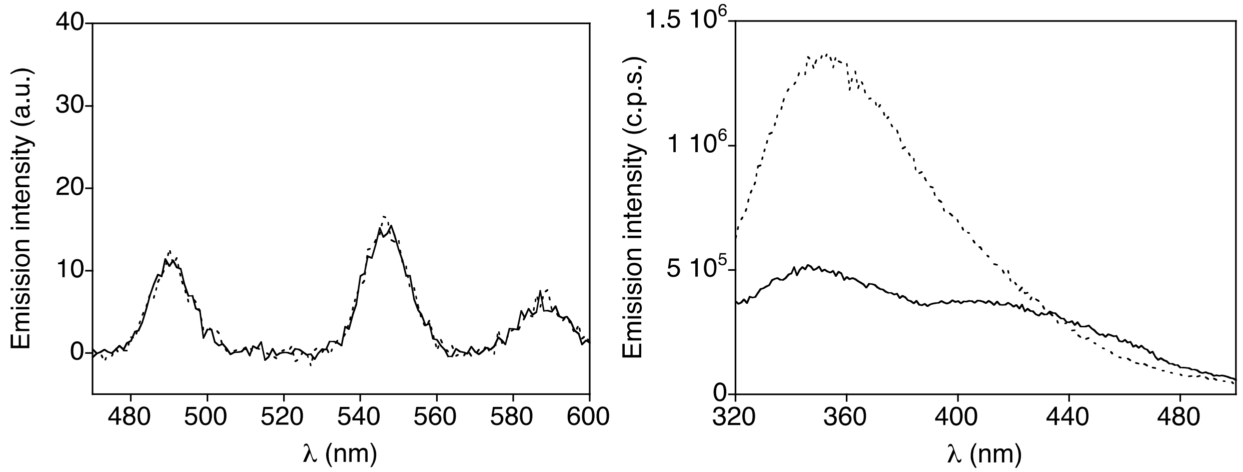

Figure S7. Left and right: Time-gated (excitation 300 nm) and steady-state (excitation 280 nm) luminescence measurements of a 100 nM solution of the peptide **W-O1Pen-Tat-K(DOTA [Tb])** in HEPES buffer (10 mM HEPES pH 7.6, 100 mM NaCl) (dashed line) and after addition of 1 equivalent of *TAR* RNA (solid line**)**. Similar results were obtained in the case of the peptides with other spacers.

Time-gated and steady state experiments with peptide (DOTA [Tb])-Tat-W: To 3 mL of a 100 nM solution of the peptide **(DOTA [Tb])-Tat-W** in HEPES buffer (10 mM HEPES pH 7.6, 100 mM NaCl), 1 equivalent of *TAR* RNA was added. The emission spectra were recorded before and after each addition.

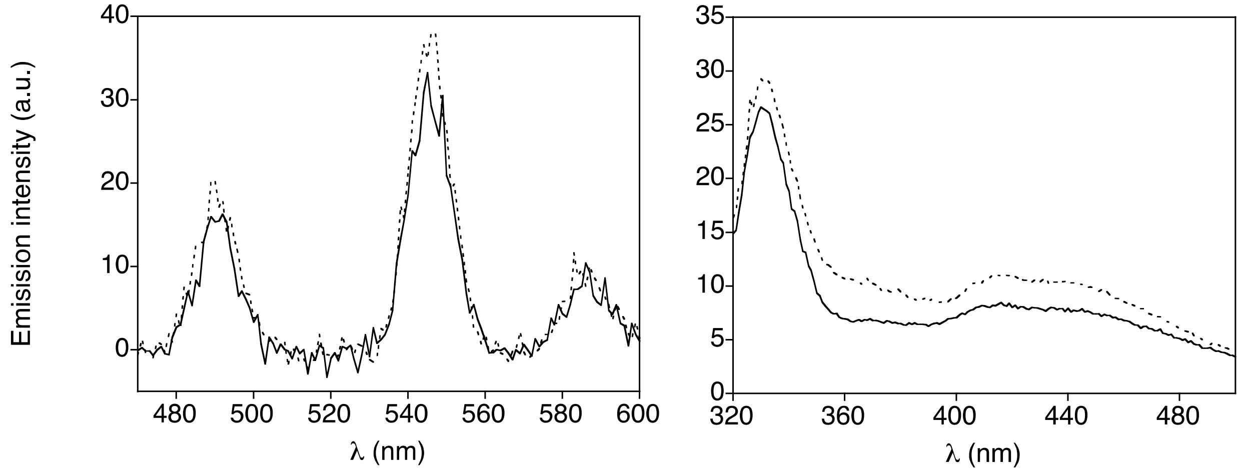

Figure S8. Left and Right: Time-gated (excitation 300 nm) and steady-state (excitation 280 nm) luminescence measurements of a 100 nM solution of the peptide **(DOTA [Tb])-Tat-W)** in HEPES buffer (10 mM HEPES pH 7.6, 100 mM NaCl) (dashed line) and after addition of 1 equivalent of *TAR* RNA (solid line)

Time-gated and steady state experiments with peptide Cou-Tat-K(DOTA[Eu]): To 3 mL of a 100 nM solution of the peptide **Cou-Tat-K(DOTA[Eu])** in HEPES buffer (10 mM HEPES pH 7.6, 100 mM NaCl), 1 equivalent of *TAR* RNA was added. The emission spectra were recorded before and after each addition.

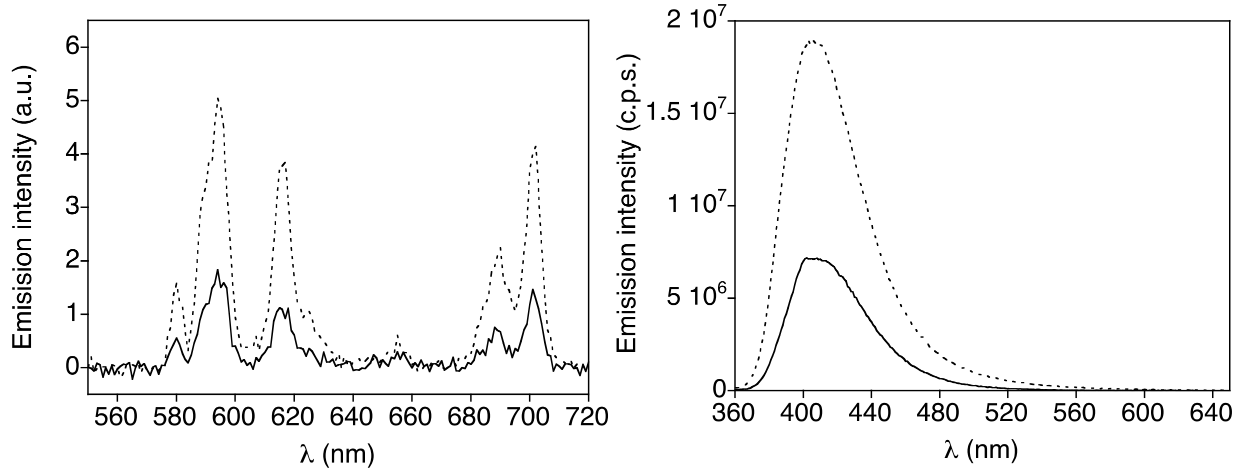

Figure S9. Left and Right: Time-gated (excitation 350 nm) and steady-state (excitation 350 nm) luminescence measurements of a 100 nM solution of the peptide **Cou-Tat-K(DOTA[Eu])** in HEPES buffer (10 mM HEPES pH 7.6, 100 mM NaCl) (dashed line) and after addition of 1 equivalent of *TAR* RNA (solid line).

Time-gated and steady state experiments with peptide Cou-P_4-Tat-P_4-K(DOTA [Eu]): To 3 mL of a 100 nM solution of the peptide **Cou-P_4-Tat-P_4-K(DOTA [Eu])** in HEPES buffer (10 mM HEPES pH 7.6, 100 mM NaCl), 1 equivalent of *TAR* RNA was added. The emission spectra were recorded before and after each addition.

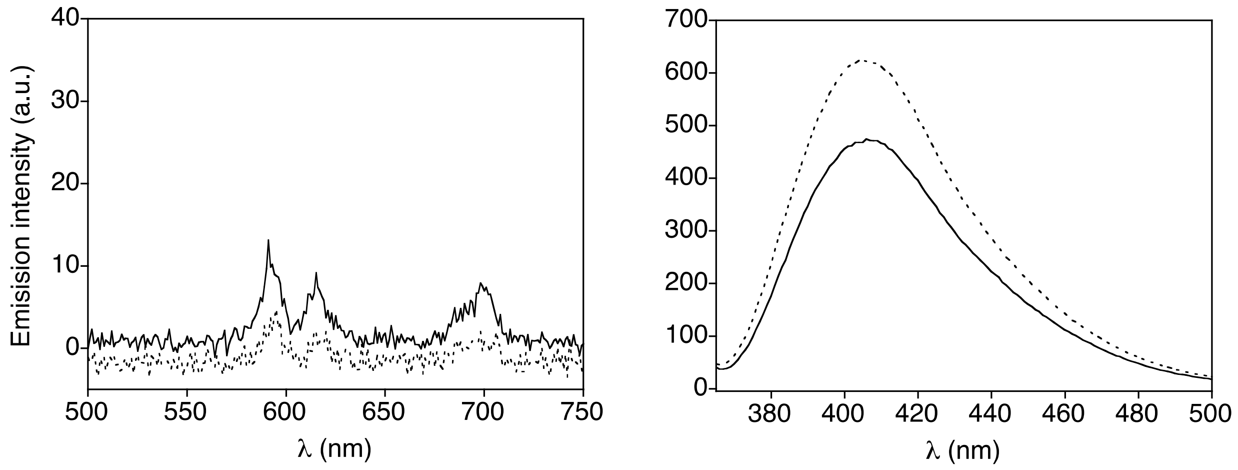

Figure S10. Left and right: Time-gated (excitation at 350 nm) and steady-state (excitation 350 nm) luminescence measurements of a 100 nM solution of the peptide **Cou-P_4-Tat-P_4-K(DOTA [Eu])** in HEPES buffer (10 mM HEPES pH 7.6, 100 mM NaCl) (dashed line) and after addition of 1 equivalent of *TAR* RNA (solid line).

Time-gated and steady state experiments with peptide TO-Tat-K(DOTA [Eu]): To 3 mL of a 100 nM solution of the peptide **TO-Tat-K(DOTA [Eu])** in HEPES buffer (10 mM HEPES pH 7.6, 100 mM NaCl), 1 equivalent of *TAR* RNA was added. The emission spectra were recorded before and after each addition.

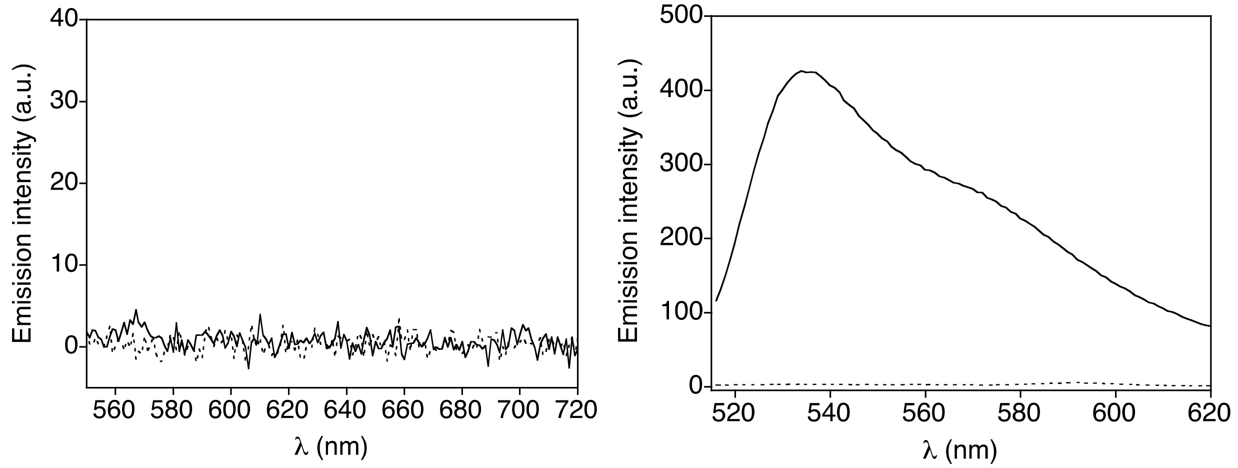

Figure S11. Left and right: Time-gated (excitation 495 nm) and steady-state (excitation 495 nm) luminescence measurements of a 100 nM solution of the peptide **TO-Tat-K(DOTA [Eu])** in HEPES buffer (10 mM HEPES pH 7.6, 100 mM NaCl) (dashed line) and after addition of 1 equivalent of *TAR* RNA (solid line). Similar results were obtained in the case of the peptide **ACR-Tat-K(DOTA[Eu])**.

4. Peptides with phenantroline antennas

4.1. Synthesis of the phenantroline derivative **8**

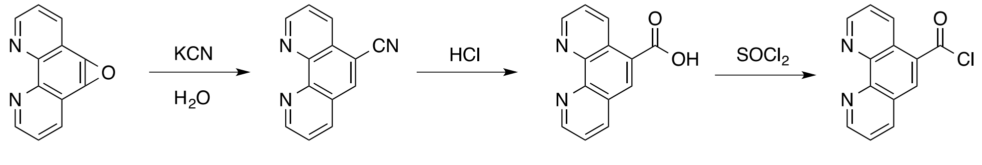

Scheme S10. Synthetic route for the preparation of the phenantroline derivative **8**.

The intermediate phenantroline **7** was synthesized as previously described.[5] Reaction of **5** (600 mg, 3.06 mmol) with potassium cyanide (1.2 g, 18 mmol) in water (aprox. 60 mL) at room temperature for 6 h affords a white precipitate that after being filtered and washed was lyophilized. In this way, 5-cyano-1,10-phenantroline (**6**) was obtained directly in an overall yield of 80%, without isolation of the intermediates. Then, **6** (aprox. 500 mg, 2.45 mmol) was smoothly hydrolyzed by refluxing in 6 M HCl for 6 days. After checking by HPLC-MS (lineal gradient 5 → 95% CH_3CN, 0.1% TFA / H_2O, 0.1% TFA in 30 min) the completion of the reaction, the crude was purified by reverse phase HPLC. The intermediate **7** was isolated as a white solid in a 69% yield (380 mg).

7: (380 mg, 69% yield)
ESI-MS (m/z): $[MH]^+$ calcd. for $C_{13}H_8N_2O_2$= 224.22, found: 225.1 $[MH]^+$.
$\mathbf{t_R}$ = 11.7 min (*Eclipse XDB-C*$_{18}$, lineal gradient 5→95% CH_3CN, 0.1% TFA / H_2O, 0.1% TFA in 30 min).

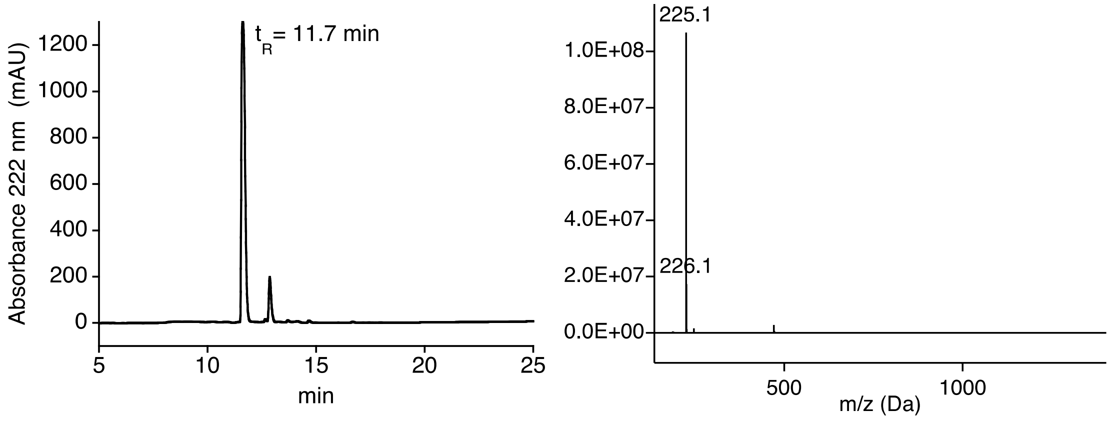

Figure S12. *Left*: HPLC trace of the pure product **7**. *Right*: ESI-MS spectra of **7**.

The molar extinction coefficient of compound **7** was obtained by linear regression analysis of the UV absorption values of samples of known concentrations by weight. ε_{278nm} = 33,847 $M^{-1}cm^{-1}$ was found for the phenanthroline derivative **7**.

7 (125 mg, 0.56 mmol) was refluxed in $SOCl_2$ (700 μL) for 5 h. Then, solvents were removed under reduced pressure. The product obtained (**8**) was used without further purification.

5 Y. Shen, B.P. Sullivan, *Inorg. Chem.*, 1995, **34**, 6235–6236.

4.2. Synthesis of peptide EDTA[Tb]-Tat-Lys(ϕ)

The synthesis of **EDTA-Tat-Lys(ϕ)** peptide was carried out following the tactic described below, in which the phenanthroline unit was inserted in the side chain of a C-terminal Lys residue, which is introduced with its side chain protected with an Mtt group. Orthogonal deprotection of this group, while the residue is still attached to the solid support, allowed the introduction of the phenantroline unit.

Following the derivatization with the phenanthroline antenna, the rest of the peptide was assembled in solid phase and finally, the chelating ethylenediaminetetraacetic acid (EDTA) unit in the form of a dianhydride was attached to the N-terminus of the peptide to give the desired peptide **EDTA-Tat-Lys(ϕ)**.

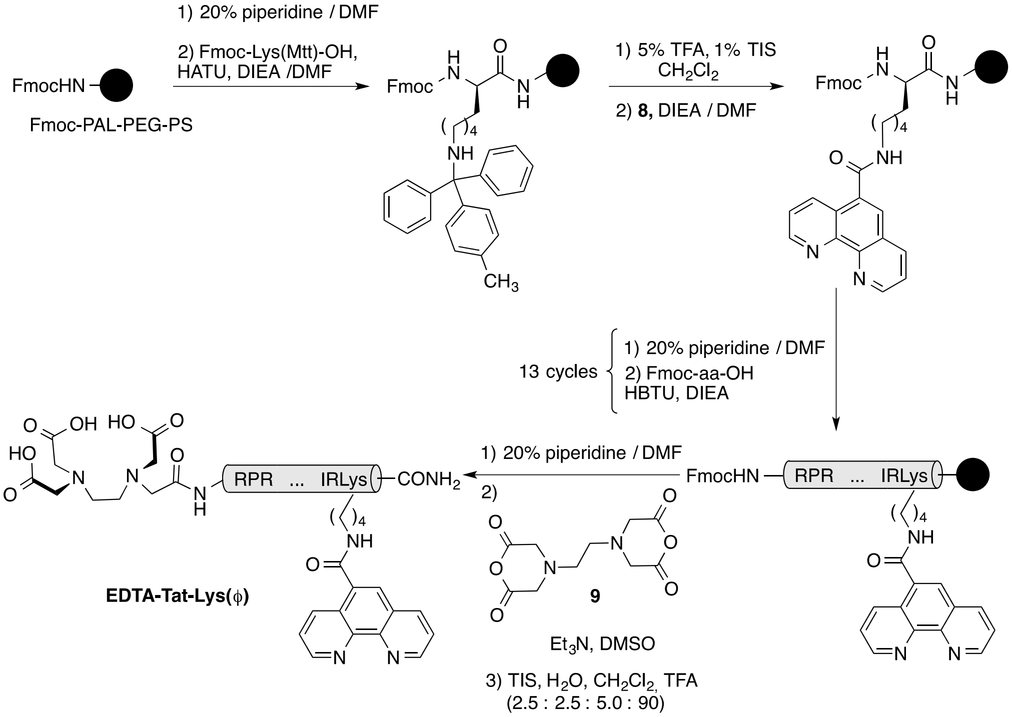

Scheme S11. Synthethic route for the synthesis of peptide **EDTA-Tat-Lys(ϕ)**.

Fmoc-Lys(Mtt)-OH coupling and orthogonal Mtt deprotection: Fmoc-Lys(Mtt)-OH was manually coupled to the resin (0.05 mmol scale using as solid support the Fmoc-PAL-PEG-PS resin). The coupling was checked by a TNBS test.[6] Then, side chain of the Lys(Mtt) residue was selectively deprotected for specific attachment of the phenanthroline unit: the resin-bound peptide (≈ 0.05 mmol) was treated three times with a mixture of TFA (250 μL), triisopropylsilane (50 μL) and CH_2Cl_2 (4.7 mL) for 2 min. The resin was then filtered and washed with CH_2Cl_2 (2 × 5 mL × 3 min).

Phenantroline coupling to the Lys side chain: **8** (62.4 mg, 0.26 mmol) was dissolved in dry DMF (470 μL). *N,N*-diisopropylethylamine (DIEA) (146 μL , 0.85 mmol) was added to the solution and resulting mixture was added over the Mtt-deprotected peptide attached to the resin (≈ 0.05 mmol). N_2 was passed through the resin suspension for 1 h and was then filtered and washed with DMF (2 × 5 mL × 3 min).

EDTA dianhydride coupling: After peptide chain elongation, the Fmoc group was deprotected by treatment with 20% piperidine in DMF (5 mL × 20 min). The resin was then filtered and washed with DMF (2 × 5 mL × 3 min). The commercial product ethylenediaminetetraacetic dianhydride (**9**, 128 mg, 0.5 mmol) was dissolved in DMSO (25 mL), and triethylamine (8 μL, 0.055 mmol) was added over the

6 W.S. Hancock, J.E. Battersby, *Anal. Biochem.,* 1976, **71**, 260.

solution. Then, the mixture was added over the resin (≈ 0.05 mmol) and the suspension was shaken overnight. The resin was then washed with DMSO (2 × 5 mL × 3 min) and CH_2Cl_2 (2 × 5 mL × 3 min).

Cleavage and deprotection of semipermanent protecting groups: The resin-bound peptide was subjected to cleavage and deprotection by treatment with the cleavage cocktail under the conditions already described. After reverse-phase HPLC purification and lyophilization of the collected fractions, the desired peptide **EDTA-Tat-Lys(ϕ)** was isolated as white solid in a 10% yield.

EDTA-Tat-Lys(ϕ): (12.5 mg, aprox. 10% for a 0.05 mmol scale)
ESI-MS (m/z): $[MH]^+$ calcd. for $C_{92}H_{152}N_{38}O_{24}$= 2174.47, found 1144.6 $[MH_2]^{2+}$ + 1 TFA, 763.2 $[MH_3]^{3+}$ + 1 TFA, 725.3 $[MH_3]^{3+}$, 544.2 $[MH_4]^{4+}$, 435.5 $[MH_5]^{5+}$, 363.1$[MH_6]^{6+}$.
$\mathbf{t_R}$ = 12 min (*Eclipse XDB-C_{18},* lineal gradient 5→95% CH_3CN, 0.1% TFA / H_2O, 0.1% TFA in 30 min).
UV (H_2O) λ_{max}: 278 nm; ε: 33,847 $M^{-1}cm^{-1}$.

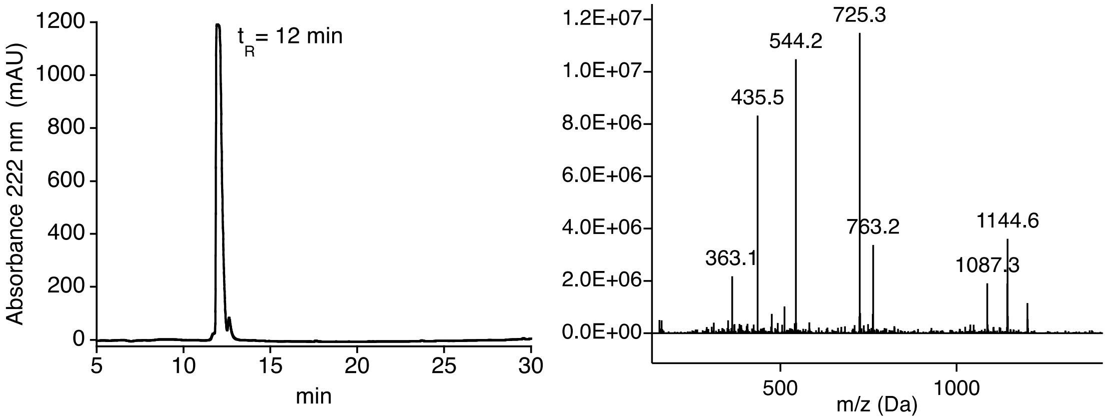

Figure S13. *Left*: HPLC trace of the pure peptide **EDTA-Tat-Lys(ϕ)**. *Right*: ESI-MS spectra corresponding to the peak at c.a. 12 min.

Analysis of EDTA*-Tat-Lys(ϕ)/Tb(III) and* EDTA*-Tat-Lys(ϕ)/Eu(III) mixtures by MS.*

Concentrated mixtures of the peptide **EDTA-Tat-Lys(ϕ)** in presence of 1 equivalent of Eu(III) or Tb(III) ions were analyzed by ESI-MS. The spectrum of a mixture of **EDTA-Tat-Lys(ϕ)** and 1 equivalent of Eu(III) is shown in the figure below, where the expected $[MH_5]^{5+}$ and $[MH_6]^{6+}$ peaks, corresponding to the lanthanide complex **EDTA-Tat-Lys(ϕ)**/Eu(III), are observed. Likewise, the analysis of the mixture of **EDTA-Tat-Lys(ϕ)** and 1 equivalent of Tb(III) displayed the expected peaks at 466.9 and 389.2 corresponding to the complex between **EDTA-Tat-Lys(ϕ)** and Tb(III).

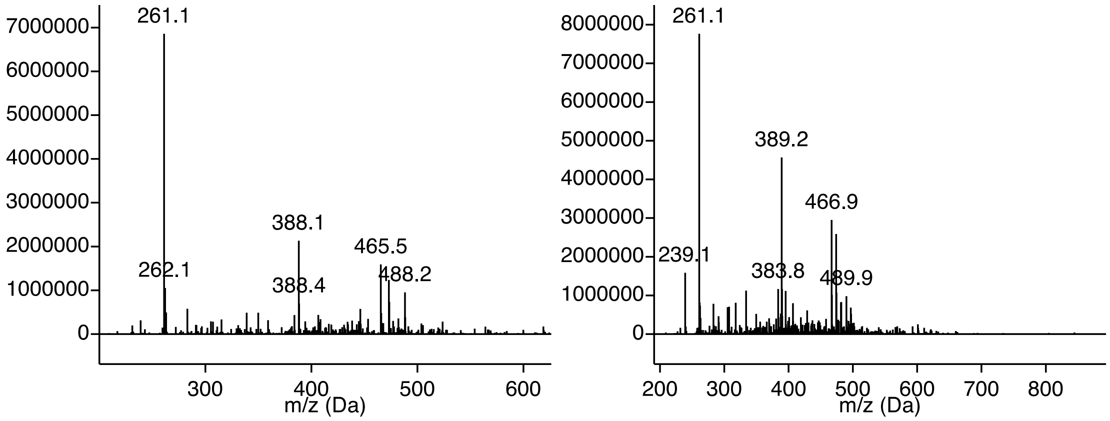

Figure S14. *Left:* Mass spectrum of an aliquot of a 40 µM solution of **EDTA-Tat-Lys(ϕ)** in the presence of 40 µM of Eu^{3+}, in 10 mM HEPES, 100 mM NaCl, pH 7.6. The peaks at 488.2, 465.5 and 388.1 correspond to the $[MH_5]^{5+}$ + 1 TFA, $[MH_5]^{5+}$ and $[MH_6]^{6+}$ ions of the lanthanide complex **EDTA-Tat-Lys(ϕ)**/Eu(III). The peak at 261.1 corresponds to the $[MNa]^+$ ion of the HEPES buffer. *Right:* Mass spectrum of an aliquot of a 40 µM solution of **EDTA-Tat-Lys(ϕ)** in the presence of 40 µM of Tb^{3+}, in 10 mM HEPES, 100 mM NaCl, pH 7.6. The peaks at 466.9 and 389.2 correspond to the $[MH_5]^{5+}$ and $[MH_6]^{6+}$ ions of the lanthanide complex **EDTA-Tat-Lys(ϕ)**/Tb(III). The peak at 261.1 and 239.1 correspond to the $[MNa]^+$ and $[MH]^+$ ions of the HEPES buffer.

4.3. Biophysical assays

Luminescence measurements

Time-gated emission titration of peptide EDTA-Tat-Lys(ϕ) with *TAR* RNA. To 3 mL of a 50 nM solution of the peptide **EDTA-Tat-Lys(ϕ)** and $EuCl_3$ or the peptide **EDTA-Tat-Lys(ϕ)** and $TbCl_3$ in HEPES buffer (10 mM HEPES pH 7.6, 100 mM NaCl), increasing amounts of *TAR* RNA were successively added. The emission spectra were recorded before and after each addition.

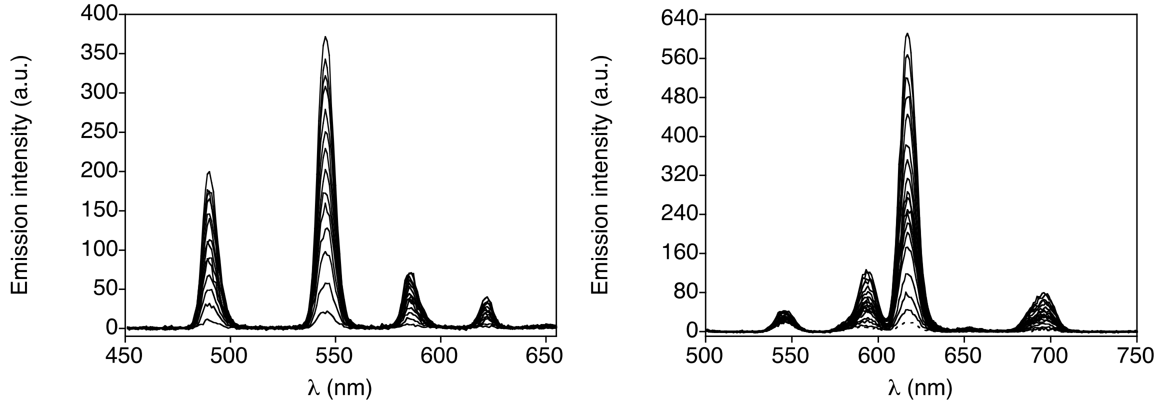

Figure S15. *Left*: Emission spectra of a 50 nM solution of the peptide **EDTA-Tat-Lys(ϕ)** and $TbCl_3$ in HEPES buffer (10 mM HEPES pH 7.6, 100 mM NaCl) (dashed line); and in presence of increasing amounts of consensus *TAR* RNA in the following concentrations: 5, 10, 15, 20, 25, 30, 35, 40, 45, 50, 55, 65, 75, 85, 125, 191, 290, 421, 520 and 617 nM (solid lines). *Right*: Emission spectra of a 50 nM solution of the peptide **EDTA-Tat-Lys(ϕ)** and $EuCl_3$ in HEPES buffer (10 mM HEPES pH 7.6, 100 mM NaCl) (dashed line); and in presence of increasing amounts of consensus *TAR* RNA in the following concentrations: 5, 10, 15, 20, 25, 30, 35, 40, 45, 50, 55, 65, 75, 85, 125, 191, 290, 421, 520 and 617 nM (solid lines).

Data fitting: The experimental data of the titration of complexes **EDTA-Tat-Lys(ϕ)**/Tb(III) and **EDTA-Tat-Lys(ϕ)**/Eu(III) with *TAR* RNA were fit to a 1:1 model for a luminescent ligand binding (**EDTA-Tat-Lys(ϕ)**/Tb(III) or **EDTA-Tat-Lys(ϕ)**/Eu(III)) to a unlabeled receptor (*TAR* RNA). If non-specific binding is ignored, then this interaction is described by the following equation, which was used to fit the experimental data using non-linear regression analysis:[7]

$$F_T = \frac{1}{2}\left(2F_0 + F_{RL} \times \left[K_D + L_T + R_T - \sqrt{\left(K_D + L_T + R_T\right)^2 - 4L_T R_T}\right]\right)$$

Where **R**, concentration of the free receptor in the equilibrium (*TAR* RNA); $\boldsymbol{R_T}$, total receptor concentration; **L**, concentration of the free labeled ligand in the equilibrium (**EDTA-Tat-Lys(ϕ)**/Tb(III) or

7 M.H.A. Roehrl, J.Y. Wang, G. Wagner, *Biochemistry,* **2004**, *43*, 16056–16066.

EDTA-**Tat-Lys(ϕ)**/Eu(III)); L_T, total concentration of the labeled ligand; K_D, dissociation constant of the interaction between the receptor and the ligand; F_T, total observed emission, F_0, adjustable parameter accounting for the background emission; F_{RL} adjustable parameter for the labeled ligand-receptor complex molar emission.

Time-gated emission of the peptide complex EDTA-Tat-Lys(ϕ)/Eu with ssDNA, dsDNA and with a non specific RNA hairpin. To 3 mL of a 50 nM solutions of peptide **EDTA-Tat-Lys(ϕ)** and $EuCl_3$ in HEPES buffer (10 mM HEPES pH 7.6, 100 mM NaCl), aliquots of a ssDNA (TGG AGA TGA CTC ATC TCG TT) or dsDNA (ds(TGG AGA TGA CTC ATC TCG TT)) were added up to 0.6 and 0.7 μM respectively and the emission spectra were recorded before and after each addition. Phage P22 *boxB* non-specific RNA hairpin (GCG CUG ACA AAG CGC) was also added over a 50 nM solution of peptide **EDTA-Tat-Lys(ϕ)** and $EuCl_3$ in HEPES buffer (10 mM HEPES pH 7.6, 100 mM NaCl) up to 0.7 μM, and the emission spectra were recorded before and after the addition.

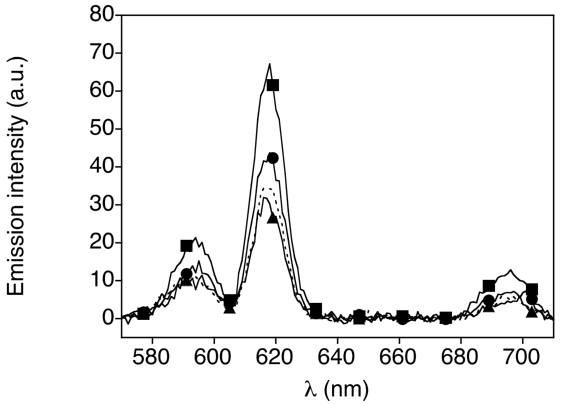

Figure S16. Emission spectra of a 50 nM solution of peptide **EDTA-Tat-Lys(ϕ)** and $EuCl_3$ in HEPES buffer (10 mM HEPES pH 7.6, 100 mM NaCl) (dashed line) and in presence of 0.6 μM ssDNA (TGG AGA TGA CTC ATC TCG TT) (■); 0.7 μM dsDNA (ds(TGG AGA TGA CTC ATC TCG TT)) (●) and 0.7 μM phage P22 *boxB* non specific hairpin RNA (▲).

Determination of the number of Tb^{3+}-coordinated water molecules for EDTA-Tat-Lys(ϕ)/Tb(III) and its complex with RNA: The emission intensity decay of the complexes **EDTA-Tat-Lys(ϕ)**/Tb(III) and **EDTA-Tat-Lys(ϕ)**/Tb(III)/*TAT* RNA is measured at 545 nm for 5 ms after an interval of 0.05 ms (room temperature). Data obtained were fitted to the exponential equation $I(t)=I(0)e(-t/\tau)$, where I(t) is the intensity at time t after the excitation pulse, I(0) is the initial intensity at time t = 0 (after the 0.05 ms interval), and τ is the luminescence lifetime. The number of water molecules in the inner coordination sphere (q) could be calculated from the lifetimes measured in water (τ_H) and deuterium oxide (τ_D) using the following empirically-derived equations:[8]

$$q(\text{Tb}) = A'(\Delta k_{corr}) \text{ where } A' = 5 \text{ ms and } \Delta k_{corr} = (1/\tau_H - 1/\tau_D - 0.06 \text{ ms}^{-1}).$$

Thus, solutions of **EDTA-Tat-Lys(ϕ)**/Tb(III) (both 50 nM in HEPES buffer -10 mM HEPES, 100 mM NaCl, pH 7.6-) and **EDTA-Tat-Lys(ϕ)**/Tb(III)/*TAT* RNA (50 nM, 50 nM and 850 nM respectively, in HEPES buffer -10 mM HEPES, 100 mM NaCl, pH 7.6-) were prepared. For the measurement in D_2O, firstly the solutions were prepared as H_2O-based buffered solutions and then those solutions were lyophilized and redissolved in the same volume of D_2O.

8 (a) R.J. Aarons, J.K. Notta, M.M. Meloni, J. Feng, R. Vidyasagar, J. Narvainen, S. Allan, N. Spencer, R.A. Kauppinen, J.S. Snaith, S. Faulkner, *Chem. Commun.,* **2006**, 909; (b) A. Beeby, I.M. Clarkson, R.S. Dickens, S. Faulkner, D. Parker, L. Royle, A.S. de Sousa, J.A.G. Williams, M. Woods, *J. Chem. Soc., Perkin Trans. 2*, **1999**, 493.

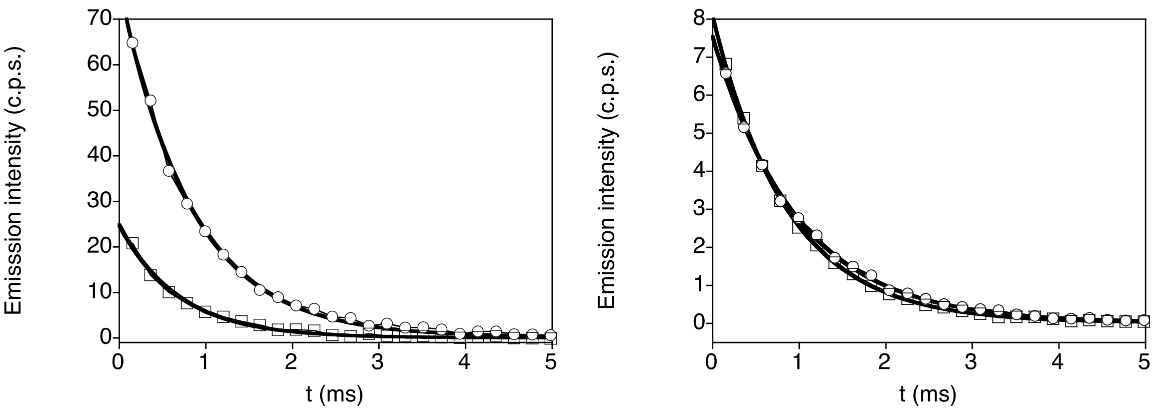

Figure S17. Luminescence decay curves of **EDTA-Tat-Lys(ϕ)**/Tb(III) (left) and **EDTA-Tat-Lys(ϕ)**/Tb(III)/*TAT* RNA (right) obtained in H_2O-based buffered solution (□) and D_2O-buffered solution (○). Concentrations of **EDTA-Tat-Lys(ϕ)**, $TbCl_3$ and *TAT* RNA are 50 nM, 50 nM and 850 nM respectively.

Table S2. Obtained values of the luminescence lifetime, in H_2O and D_2O, and number of Tb^{3+}-coordinated water molecules for **EDTA-Tat-Lys(ϕ)**/Tb(III) and **EDTA-Tat-Lys(ϕ)**/Tb(III)/*TAT* RNA.

**Compound**	1/τ **(D_2O)**	1/τ **(H_2O)**	***q***
**EDTA-Tat-Lys(ϕ)**/Tb(III)	1.20	1.45	0.95
**EDTA-Tat-Lys(ϕ)**/Tb(III)/*TAT* RNA	1.02	1.15	0.35

Similarly, the lifetimes for **EDTA-Tat-Lys(ϕ)**/Eu(III) and **EDTA-Tat-Lys(ϕ)**/Eu(III)/*TAT* RNA yielded values of 1/τ 1.78 ms, and 1.69 ms, respectively.

Time-gated emission of EDTA-Tat-Lys(ϕ) in presence of cell lysates. The luminescence spectra of a 50 nM solutions of peptide **EDTA-Tat-Lys(ϕ)** and $EuCl_3$, and peptide **EDTA-Tat-Lys(ϕ)** and $TbCl_3$ in HEPES buffer (10 mM HEPES pH 7.6, 100 mM NaCl) were measured before, and after the addition of 3.4 µL of a 147 ng/µL RNA stock solution of cell lysate (total RNA concentration approximately 170 ng/mL). The luminescence spectrum were recorded again after the addition 20 equivalents of *TAR* RNA to the **EDTA-Tat-Lys(ϕ)/Eu** and **EDTA-Tat-Lys(ϕ)/Tb** solutions in the presence of the lysate.

Preparation of cell lysates: Vero cells were grown in monolayers and lysed in buffer A (10 mM HEPES pH 7.2, 10 mM KCl, 100 mM NaCl, 0.5% Triton X100, and proteinase inhibitor cocktail by Sigma). Lysates were cleared by centrifugation, and their concentration was adjusted to contain approximately 147 nanograms per microliter of cellular RNA. Cellular extracts were kept on ice and immediately used for the experiments.

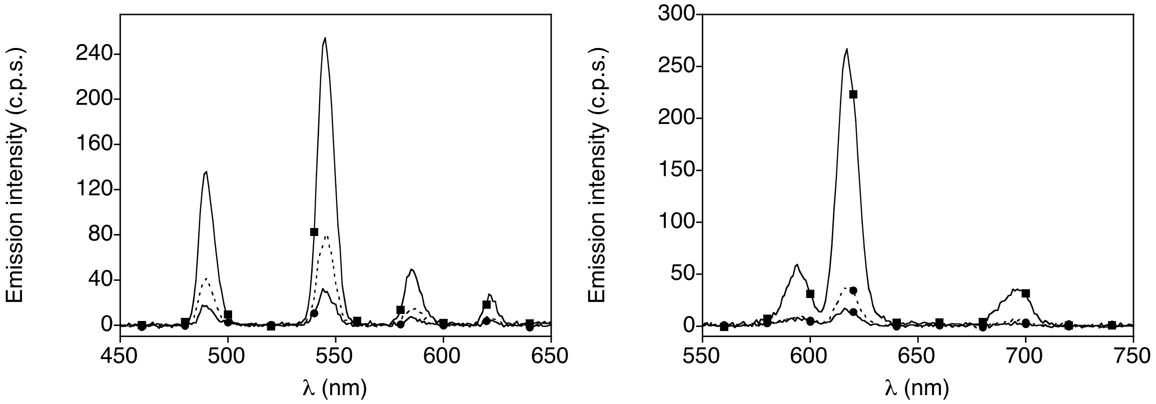

Figure S18. *Left:* Time-resolved luminescence emission of a 50 nM solution of **EDTA-Tat-Lys(ϕ)** and $TbCl_3$ in 10 mM HEPES buffer, 100 mM NaCl, pH 7.6 (dashed line); in the presence of 170 ng/mL total RNA from cell lysate (●); and after addition of 20 eq of *boxB* RNA (■). *Right*: Time-resolved luminescence emission of a 50 nM solution of **EDTA-Tat-Lys(ϕ)** and $EuCl_3$ in 10 mM HEPES buffer, 100 mM NaCl, pH 7.6 (dashed line); in the presence of 170 ng/mL total RNA from cell lysate (●); after addition of 20 eq of *boxB* RNA (■).

Time-gated emission of EDTA[TB]-Tat-Lys(ϕ) in complete cell lysates: The peptide sensor **EDTA-Tat-Lys(ϕ)** and $TbCl_3$ were added over 1mL of three different cell lysates to a final concentration of 150 nM. The lysates were prepared from HeLa cells that had previously been transfected with *TAR* RNA, *boxB* RNA, or a blank buffer solution (no exogenous RNA).

Protocol for RNA transfection and preparation of the cell lysates: Monolayers of HeLa cells were transfected at 70-90% confluency following the manufacturer's instructions and using lipofectamine® 2000 (Thermo Fisher Scientific) and 2μg of the corresponding RNA per well were used. Cells were incubated at 37ºC for 4 hours and then the solution was removed. After washing each well twice with 10 mM HEPES pH 7.4, 10 mM KCl, 100 mM NaCl buffer, cells were lysed with 1 mL of 10 mM HEPES pH 7.4, 10 mM KCl, 100 mM NaCl, 0.5% Triton X100 buffer. Lysates were cleared by centrifugation, and kept on ice and immediately used for the experiments.

Calculation of the detection limit. 20 replicates of a blank sample (50 nM solution of **EDTA-Tat-Lys(ϕ)** and $EuCl_3$) were measured. The limit of detection was calculated from the value of the mean emission intensity at 616 nm plus five times the standard deviation.

Multicolor detection experiments

All the luminescence measurements next described were time-resolved experiments, using the settings previously described for time-gated emission measurements.

The emission is normalized to those of the peptides mixture in absence of RNA. As the 592 and 614 nm Eu emissions overlap with the Tb $^5D_4 \longrightarrow ^7F_J$ bands (J = 4, 586 nm; J = 3, 621 nm), we monitored the $^5D_0 \longrightarrow ^7F_3$ (655nm) Eu band, to which the Tb contribution is negligible.[9]

Detection of ***boxB*** RNA hairpin: To a mixture of the peptide probes **P22-N^W[Tb]** and **EDTA-Tat-Lys(ϕ)** –both 50 nM– in presence of $EuCl_3$ 50 nM in HEPES buffer (HEPES 10 mM, NaCl 100 mM, pH 7.6), 1 equivalent of *boxB* RNA was added. The emission spectra were recorded before and after the RNA addition. Error bars show standard error based on three independent experiments.

Detection of ***TAR*** RNA hairpin: To a mixture of the peptide probes **P22-N^W[Tb]** and **EDTA-Tat-Lys(ϕ)** –both 50 nM– in presence of $EuCl_3$ 50 nM in HEPES buffer (HEPES 10 mM, NaCl 100 mM, pH 7.6), 1

9 S.V Eliseeva, J.-C.G. Bünzli, *Chem. Soc. Rev.*, **2010**, *39*, 189–227.

equivalent of *TAR* RNA was added. The emission spectra were recorded before and after the RNA addition. Error bars show standard error based on three independent experiments.

Detection of both ***boxB*** and ***TAR*** RNA hairpins: To a mixture of the peptide probes **P22-N^W[Tb]** and **EDTA-Tat-Lys(ϕ)** –both 50 nM– in presence of $EuCl_3$ 50 nM in HEPES buffer (HEPES 10 mM, NaCl 100 mM, pH 7.6), 1 equivalent of *boxB* RNA and 1 equivalent of *TAR* RNA were successively added.[10] The emission spectra were recorded before and after each addition. Error bars show standard error based on six independent experiments.

Determination of luminescence quantum yields

The luminescence quantum yields were calculated following already described procedures[11], using equation (1), were F is the integral photon flux, f is the absorption factor, η is the refractive index of the solvent and Φ is the quantum yield. S denotes de sample and R denotes the standard. An aqueous air-equilibrated solution of $[Ru(bpy)_3]Cl_2$ (Φ = 0.028) was used as a reference (R) compound.[12]

$$\Phi_S = \Phi_R \times \frac{F_R \times \eta^2{}_S \times f_S}{F_S \times \eta^2{}_R \times f_R} \qquad (1)$$

Isoabsorbant aqueous-buffered solutions of **EDTA-Tat-Lys(ϕ)**/Eu(III) and **EDTA-Tat- Lys(ϕ)**/Tb(III) and the $[Ru(bpy)_3]Cl_2$ standard were prepared. Their emisssion was measured using the same settings (excitation wavelenght 266 nm; excitation slit width 5.0 nm, emission slit width 10.0 nm; increment 1.0 nm; average time 0.2 s; PMT detector voltage 600 V).

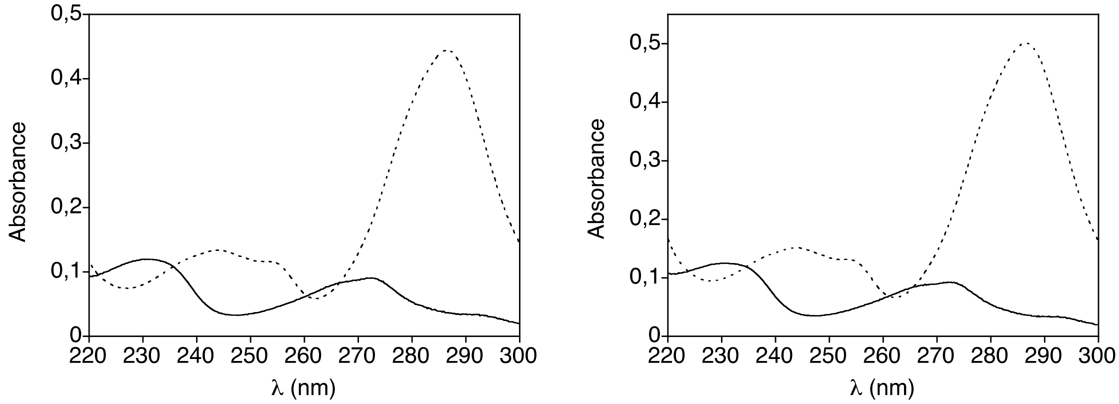

Figure S19. Representative UV spectra of aqueous **EDTA-Tat-Lys(ϕ)**/Ln(III) complexes (solid lines) and the standard ($[Ru(bpy)_3]Cl_2$ (dashed lines). *Left:* **EDTA-Tat-Lys(ϕ)**/Tb(III) and $[Ru(bpy)_3]Cl_2$. *Right:* **EDTA-Tat-Lys(ϕ)**/Eu(III) and $[Ru(bpy)_3]Cl_2$.

10 Three independent experiments were performed in which the order of addition of RNA was: 1) *boxB* RNA 2) *TAR* RNA. Other three independent experiments were done varying the order of addition of both RNAs.

11 C. Würth, M. Grabolle, J. Pauli, M. Spieles and U. Resch-Genger, *Nat Protoc.*, 2013, **8**, 1535-1550.

12 (a) K. Nakamura, *Bull. Chem. Soc. Jpn.*, 1982, **55**, 2697–2705; (b) Y. Kitamura, T. Ihara, Y. Tsujimura , Y.Osawa, D. Sasahara, M. Yamamoto, K. Okada, M. Tazaki, A. Jyo, *J. Inorg. Biochem.*, 2008, **102**, 1921–1931.

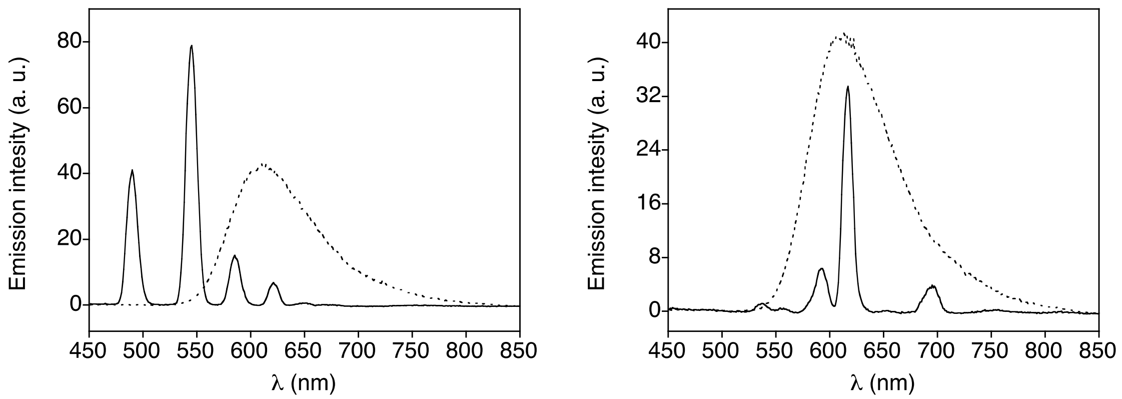

Figure S20. Representative fluorescence emission spectra of the EDTA-**Tat-Lys(ϕ)**/Ln(III) complexes (solid lines) and the standard ([Ru(bpy)$_3$]Cl$_2$, dashed lines). *Left:* EDTA-**Tat-Lys(ϕ)**/Tb(III), and [Ru(bpy)$_3$]Cl$_2$. *Right* EDTA-**Tat-Lys(ϕ)**/Eu(III) and [Ru(bpy)$_3$]Cl$_2$.

Circular Dichroism

To 300 μL of a 5 μM solution of peptide EDTA-**Tat-Lys(ϕ)** in HEPES buffer (HEPES 10 mM, NaCl 100 mM, pH 7.6), 3 μL of a 500 μM solution of Eu(III) (EuCl$_3$ 500 μM/HCl 0.1 mM) and 30 μL of a 50 μM stock solution of *TAR* RNA were successively added; and the circular dichroism spectra were recorded after the addition.

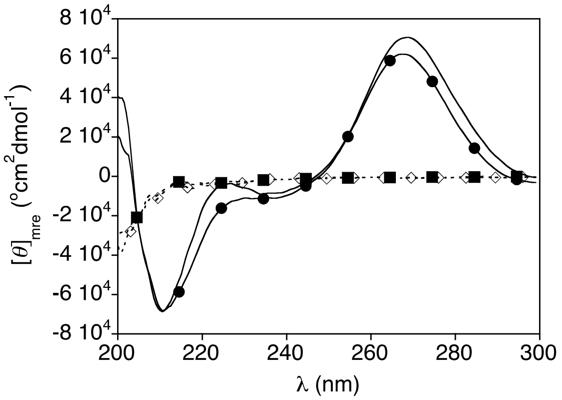

Figure S21. Dashed lines: CD of a 5 μM solution of the peptide EDTA-**Tat-Lys(ϕ)** sensor (◇) in 10 mM HEPES buffer, 100 mM NaCl, pH 7.5 and in presence of 1 equiv of Eu^{3+}(□). Solid lines: CD of a 5 μM solution of the *TAR* RNA in 10 mM HEPES buffer, 100 mM NaCl, pH 7.5 (solid line), and in the presence of 1 equiv of peptide EDTA-**Tat-Lys(ϕ)** sensor and 1 equiv of EuCl$_3$ (●).

EMSA experiments

Binding assays of EDTA-Tat-Lys(ϕ) and EDTA-Tat-Lys(ϕ)/Eu(III) **with *TAR* RNA:** Increasing concentrations of the natural peptide **Tat**, the peptide sensor EDTA-**Tat-Lys(ϕ)** and the lanthanide complex EDTA-**Tat-Lys(ϕ)**/Eu(III) were incubated with the target RNA *TAT* (5'-GGC UCG UGU AGC UCA UUA GCU CCG AGC C-3') for 20 min and analysed by EMSA.

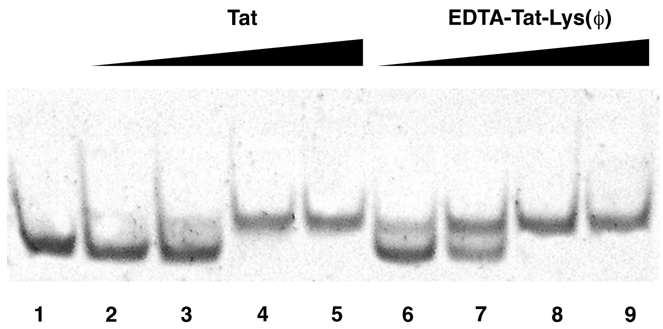

Figure S22. PAGE of *TAR* RNA in presence of peptides **Tat** and **EDTA-Tat-Lys(ϕ)**. Lanes 2, 3, 4 and 5: 50, 100, 500 and 1000 nM of **Tat**; lanes 6, 7, 8 and 9: 50, 100, 500 and 1000 nM of **EDTA-Tat-Lys(ϕ)**. All lanes contain 150 nM *TAT* RNA in 20 mM Tris·HCl pH 7.5, 90 mM KCl, 1.8 mM $MgCl_2$, 1.8 mM EDTA, 9% glycerol, 0.11 mg/mL BSA, 2.25% NP-40.

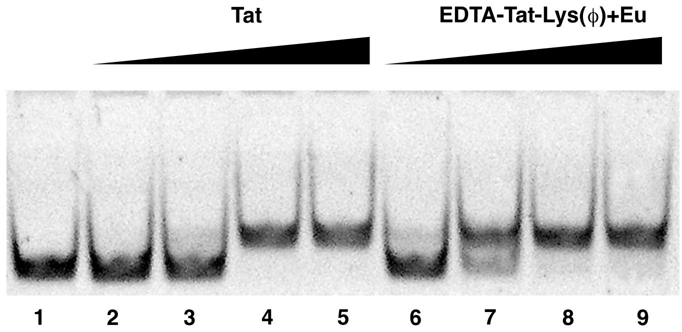

Figure S23. PAGE of *TAR* RNA in presence of peptides **Tat** and **EDTA-Tat-Lys(ϕ)** + Eu(III). Lanes 2, 3, 4 and 5: 50, 100, 500 and 1000 nM of **Tat**; lanes 6, 7, 8 and 9: 50, 100, 500 and 1000 nM of **EDTA-Tat-Lys(ϕ)**. All lanes contain 150 nM *TAT* RNA in 20 mM Tris·HCl pH 7.5, 90 mM KCl, 1.8 mM $MgCl_2$, 1.8 mM EDTA, 9% glycerol, 0.11 mg/mL BSA, 2.25% NP-40.
